# An in-situ small angle x ray scattering analysis of nanopore formation during thermally induced chemical dealloying of brass thin foils

**DOI:** 10.1038/s41598-018-33787-z

**Published:** 2018-10-18

**Authors:** Bao Lin, Max Döbeli, Stephen Mudie, Adrian Hawley, Peter Hodgson, Lingxue Kong, Ralph Spolenak, Ludovic F. Dumée

**Affiliations:** 1 Deakin University, Geelong, Institute for Frontier Materials, Waurn Ponds, 3216 Victoria, Australia; 2 ETH Zurich, Vladimir-Prelog-Weg 5, 8093 Zürich, Switzerland; 3 Australian Synchrotron, Clayton, 3168 Victoria, Australia

## Abstract

The development of non-noble nano-porous metal materials is hindered by surface oxidation reactions and from the difficulty to generate long range order pore arrays. Dealloying is a promising route to generate such materials by selective chemical etching of metal alloy materials. This process can generate nano-metal materials with superior plasmonic, catalytic and adsorptive surface properties. Here, the impact of properties of the etching solution on the dealloying process to generate nano-pores across thin film alloys was investigated by *in-situ* SAXS dealloying experiments. Single phase CuZn alloys were used as model materials to evaluate the influence of the solution temperature on the pore formation kinetics. This novel analysis allowed to visualize the change in surface properties of the materials over time, including their surface area as well as their pore and ligament sizes. The dealloying kinetics at the very early stage of the process were found to be critical to both stable pore formation and stabilization. SAXS *in-situ* data were correlated to the morphological properties of the materials obtained from *ex-situ* samples by Rutherford back scattering and scanning electron microscopy.

## Introduction

Nano-structured metals offer great potential for bio-sensing, photonics and catalysis applications^1^. The high surface to volume ratio of nano-textured metals exposes a high density of grain edges and crystallite planes offering advanced plasmonic properties which may be utilised to generate surface responsive materials. An approach to generate such surfaces is to design nano-pores across metal materials through either bottom-up or top-down strategies^1^. Amongst the different options, selective chemical dealloying is a simple etching top-down process that has recently been demonstrated for the fabrication of porous metal materials^1–6^. Selective dealloying is essentially a controlled corrosion process, naturally happening for metals exposed to environments and has been reported for a number of alloys^7,8^, such as copper alloy^8–11^, gold alloy^12–14^ or silver alloy^15^.

Dealloyed materials with pore sizes from 3 nm to 5 μm^16^ and porosities in the range of 20~70%^17–19^ have been produced, demonstrating the breadth of potential micro- to nano- structures achievable. These materials exhibit unique pore morphologies generated by an intricate series of continuous 3D metal bridges, called ligaments. The pore size distribution, porosity and pore interconnectivity may be tuned by varying material properties and etching conditions^6^. Indeed, the alloy composition, the number of phases and the material micro-structure will greatly affect the dynamics of the dealloying process including pitting initiation on the metal surface and pore propagation^2^. Altering the processing parameters such as the etchant concentration, etchant nature, and residence time for chemical dealloying, and the potential difference for electro-chemical dealloying will also affect the kinetics of dealloying. Changes in the kinetics result in changes to the reorganization of the atoms locally displaced by the removal of specific metal atoms from the grain crystals^18^. The effective thickness of the materials may also lead to localized pore size distributions and porosity variations due to polarization concentration gradients of metal ions formed across the newly formed pores^17^. Despite this unavoidable phenomenon, porous materials with thicknesses from hundreds of nanometres up to millimetres were previously successfully formed by selective dealloying^4^.

The dealloying process is based on surface interactions between metal atoms across surface grains of an alloyed material and etchant molecules in solution coming in contact with the metal surface^20^. Redox reactions on the surface of the metal alloy will lead to localized oxidation of metal into metallic ions, which will be released in solution^21^. The vacancies created by the metal migration will lead to local rearrangements between the most noble atoms remaining in the structure to reduce the surface energy of the material and reform into crystalline structures, leading to pore formation^7,17^. The dealloying of an ideal, atomically smooth, and crystalline surface will therefore start from localized surface pitting across the metal grains with fewer noble atoms. Pitting will enhance the specific surface area of the material and generate vacancies^22^ across the surface which will propagate through the thickness of the material, creating islands of noble metal^18^. The average ligament size was found to be related to the process temperature and alloy composition^23–25^. The duration of the dealloying treatment will therefore primarily be thickness and composition related, with recent works on nano-porous copper suggesting that complete dealloying of 1 to 2 µm may be achieved in timescales on the order of minutes to tens minutes. However, the ligament size was shown to change even after dealloying due to further re-arrangements of noble atoms between ligaments in order to reduce the surface energy of the metal matrix^14,26^. Although nano-porous copper materials offer promising properties, better understanding of their formation mechanisms and surface reactivity is strongly needed to custom engineer the next generation of catalyst, electrode, and sensor^24,27–30^ materials.

In this paper, *in-situ* Small Angle X-ray Scattering (SAXS) patterns, acquired at the Australian Synchrotron during Cu-Zn dealloying experiments, were used to investigate the kinetics of development of Cu dealloyed nano-structures. SAXS patterns corresponding to these intricate network structures were used to evaluate the distribution and polydispersity of the pores and to correlate to surface roughness of the materials, both in terms of film and pore surfaces^31^. These structural features were correlated to scanning electron micrographs (SEM) while the compositions of the alloys at different dealloying durations were evaluated by Proton-Induced X-ray Emission (PIXE) analysis, allowing for non-destructive probing of the materials in a depth of microns^32^. In addition, Rutherford Back-Scattering (RBS) was used to reveal relative location of the elements, which was useful to detect the oxide layer formed during the passivation of the copper outer shells of the materials upon etching. In such a way, SAXS was used to critically evaluate kinetics of pore formation while RBS provided direct evidence of kinetics of both thickness variations and oxidation levels. This approach provides the first comprehensive physical-chemical analysis of dynamic de-alloying experiments and paves the ways to novel alloy designs towards controlled formation of nano-pores in metals.

## Materials and Methods

### Materials and chemicals

The brass films (CuZn30) of 25 µm in thickness, were purchased from Goodfellow^®^, UK. Sodium Hydroxide (NaOH, reagent grade, pellets, provided by Sigma-Aldrich^®^) was used for etching. Deionized water was used to prepare the etching solutions. All chemicals and films were used as received without further treatments. The characterisation of pristine brass films was shown in the Supplementary Materials: S2.

### Characterization techniques

Scanning Electron Microscopy (SEM) was performed on a Supra 55VP FEG SEM (Zeiss, Germany) at 5 kV for a 10-mm working distance. The samples were not coated prior to imaging and used as dealloyed. Focus Ion Beam (FIB) cross sections were performed on a Quanta 3D FEG FIB-SEM (FEI, USA) with a Ga liquid metal ion source at 30 kV and a 10-mm working distance. The detailed information on the milling process was shown in the Supplementary Information.

Proton-Induced X-ray Emission (PIXE) and Rutherford Back Scattering (RBS), are two non-destructive materials characterisation techniques allowing for elemental analysis. The dealloyed samples were cut into squares (1 cm × 1 cm) and directly used in transmission. RBS analysis was performed with 2 MeV ^4^He beam and a silicon PIN diode detector at a scattering angle of 168°. The depth resolution was on the order of 10 to 20 nm^33^. Flat background was subtracted using a universal fitting procedure^34^ and the data was analysed with the RUMP software (v.14)^35^. PIXE spectra were acquired simultaneously using the same 2 MeV He beam. Emitted X-rays were collected with a silicon drift diode detector.

A Bruker Wyko NT1100 profilometer (Bruker, USA) was used to perform batch surface roughness measurements on the dealloyed samples. The measurement was performed with standard scanning speed on an area of approximately 22,500 μm^2^, corresponding to a relatively large but yet flat area in the field of view. The roughness *R*_a_ was calculated by measuring 5 different areas and then taking the average roughness of these sampled areas.

SAXS experiments were performed at the Australian Synchrotron (AS, Melbourne, Australia) on the SAXS/WAXS beamline. The scattering patterns were analysed with Scatterbrain 2.10 (Melbourne, Australia) supplied from the SAXS group at the AS following the procedures previously described^36^. The detector was a Pilatus 1 M (Melbourne, Australia) with a beam energy of 16 keV. The acquisition of the SAXS patterns was performed every 30 s. The camera was set at 7 m which revealed features between 7.5~450 nm.

### *In-situ* dealloying test on SAXS

*In-situ* chemical dealloying experiments were performed with a quartz flow cell acting as a dealloying reactor, in which 1 cm^2^ of the sample was exposed to an etching electrolyte shear flow on one side only. The schematic of the quartz flow cell and the set-up of *in-situ* experiment is shown in supplementary materials S2. The etching solution was placed into a jacketed beaker and equilibrated for 30 min prior to the experiments at the target temperature of 5, 20, 40 or 60 °C. The samples were attached to the flow cells with double sided Kapton® tape to seal the system and prevent liquid leakages. An extra layer of Kapton® tape was placed on the back of the sample, outside the quartz cell, to ensure that the etching solution would not leak. The samples were put vertically in the pathway of the X-rays and facing the detector and the etching solution side faced the beam source. Only one side of the samples was therefore dealloyed at a time. The etching solution was pumped through the flow cell at a flow rate of 30 mL∙min^−1^. Patterns acquired just before the dealloying solution contacted the samples were used as a background. Each sample was therefore its own reference and the physical area probed by the X-ray beam was consistently the same during each test. A schematic of the process is given in Figure S1(c). The flow cell used for the *in-situ* dealloying tests was composed of four layers of materials across the X-ray beam pathway, including 1 quartz wall, the solution layer, dealloyed material, and pristine material. An empty shot on the cell itself was used as the background for these experiments.

### SAXS analysis and modelling

The dealloyed material was considered as an amorphous structure^31^, which did not present characteristic peaks but a broad knee instead. The formation of Cu-Zn porous structure fabricated in alkali solution was relatively slow when compared with previous studies^4,37^, requiring a duration of more than 24 h. In this paper, the metal sheet was considered as a homogenous medium, while the formation and development of dealloyed features/pores were thought as the formation of second phase in dilution solution. Therefore, basic parameters of the SAXS pattern, such as radius of gyration (*R*_g_), scattering volume (*V*_s_), and surface area (*A*), were measured to reveal the kinetics of early stage of Cu-Zn dealloying system. A detailed description of the modelling is shown in Supplementary Materials: S3.

## Results and Discussion

Initially *ex-situ* analysis was performed on a series of pre-etched samples under different conditions to understand the impact of the temperature of the dealloying bath on morphology and composition of the ligaments formed. Then *in-situ* SAXS analysis was performed on fresh samples to reveal bulk morphological changes and pore size distributions upon dealloying.

### Morphology and impact of dealloying temperature and duration

The impact of dealloying conditions was investigated by comparing the morphology of dealloyed samples under a range of experimental conditions. The morphology of dealloyed CuZn films was altered significantly depending on the etching duration and solution temperature. Granulous copper oxide precipitates that formed the ligaments appeared on the surface of dealloyed CuZn samples at every temperature probed.

**Figure 1:**
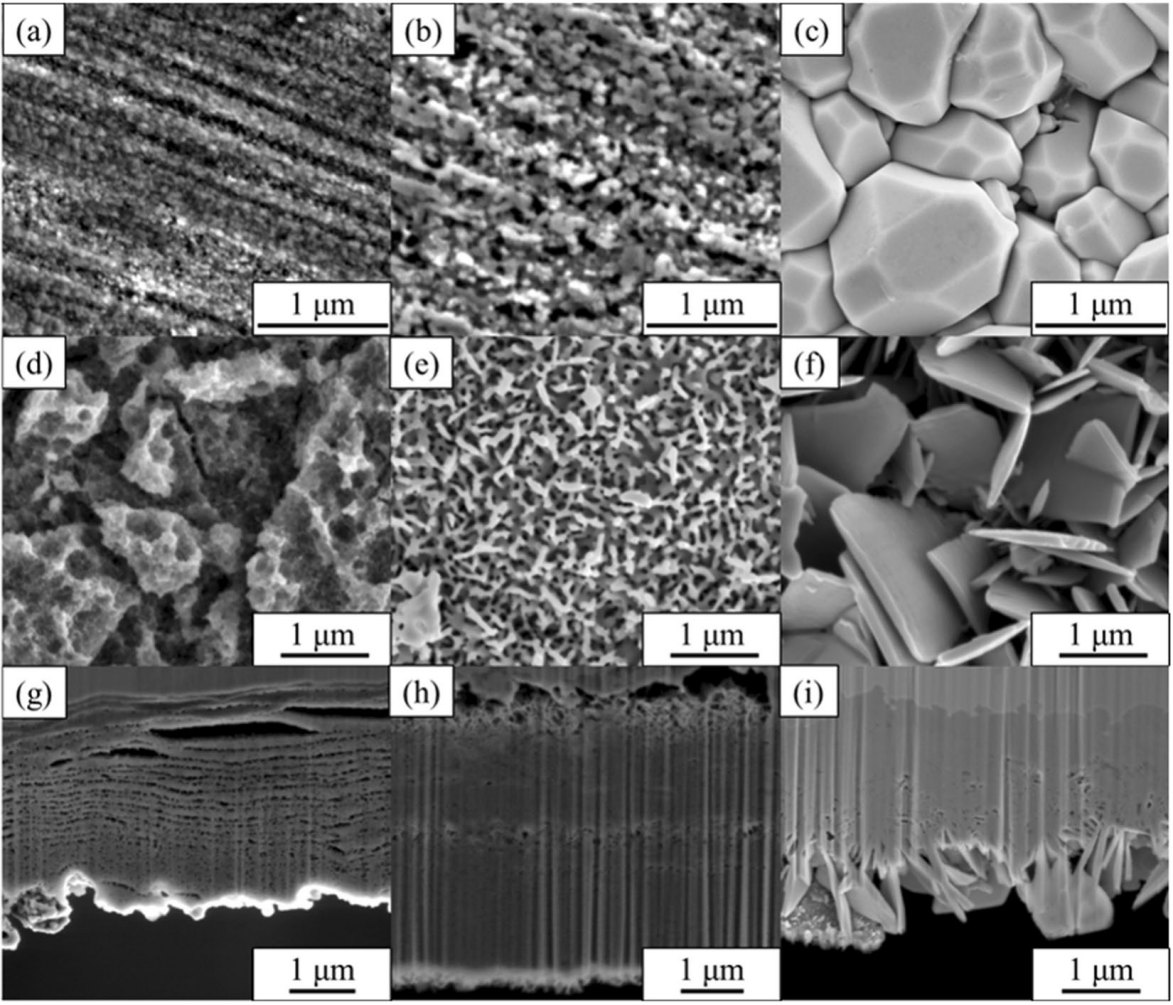
The morphology of brass de-alloyed with 1 M NaOH. (**a**) De-alloyed at 5 °C for 1 h. (**b**) De-alloyed at 20 °C for 1 h. (**c**) De-alloyed at 60 °C for 1 h. (**d**) De-alloyed at 5 °C for 96 h. (**e**) De-alloyed at 20 °C for 96 h. (**f**) De-alloyed at 60 °C for 96 h. (**g**) Cross-section view of (**d**). (**h**) Cross-section view of (**e**). (**i**) Cross-section view of (**f**). All cross-section views were taken from 52° tilted samples.

It was found that dealloying of the Cu-Zn system led to granulous ligament nano-structures (Fig. 1(a–f)). Over the course of the first hour treatment, the average diameter of the precipitates was however found to change proportionally with both an increasing solution temperature and duration. After 96 h of dealloying, the dealloyed structures generated at 5 °C exhibited ligament structures on the order of 20 nm while at 20 °C the average width reached 120 ± 40 nm. The precipitates at 60 °C converted into flaky structure rather than porous framework due to the increase of diffusion rate of Cu ions at 60 °C. This changes may be related to the activation energies of Zn in either bulk Cu or the less crystalline grain boundaries^38^.

Additionally, a series of thin porous layers were formed across the dealloyed Cu-Zn samples at every temperature (Fig. 1(g–i)). These nano-layers, with thickness ranging from below 100 nm to over 5 μm, may have been propagated by delamination of dealloyed material due to release of mechanical stress upon dealloying. Indeed, the dealloying process leads to the formation of a thin oxide layer across the surface of metal material, which is propagated by selective etching and atomic rearrangement of Cu atoms. The pore formation is likely to induce specific stress at the boundary between surface grains which may lead to flaking and generation of ultra-thin nano-porous metal oxide layers. The analysis of top layers of the materials is therefore critical to evaluate the dynamic progression of the dealloying mechanisms.

### Composition analysis of *ex-situ* dealloyed samples

Previous research on dealloying used residual Zn to represent the progress of de-alloying process^7,8,11,39^ by correlating residual Zn to variations in de-alloyed morphology. However, the residual Zn cannot directly reveal the progress of the de-alloying since the dissolution of Zn depends on whether Zn has been directly exposed to etching solution. The transfer of Zn atoms by Kirkendall effects may be terminated upon passivation of the surface or from a lack of vacancies providing diffusion pathways^17^.

**Figure 2:**
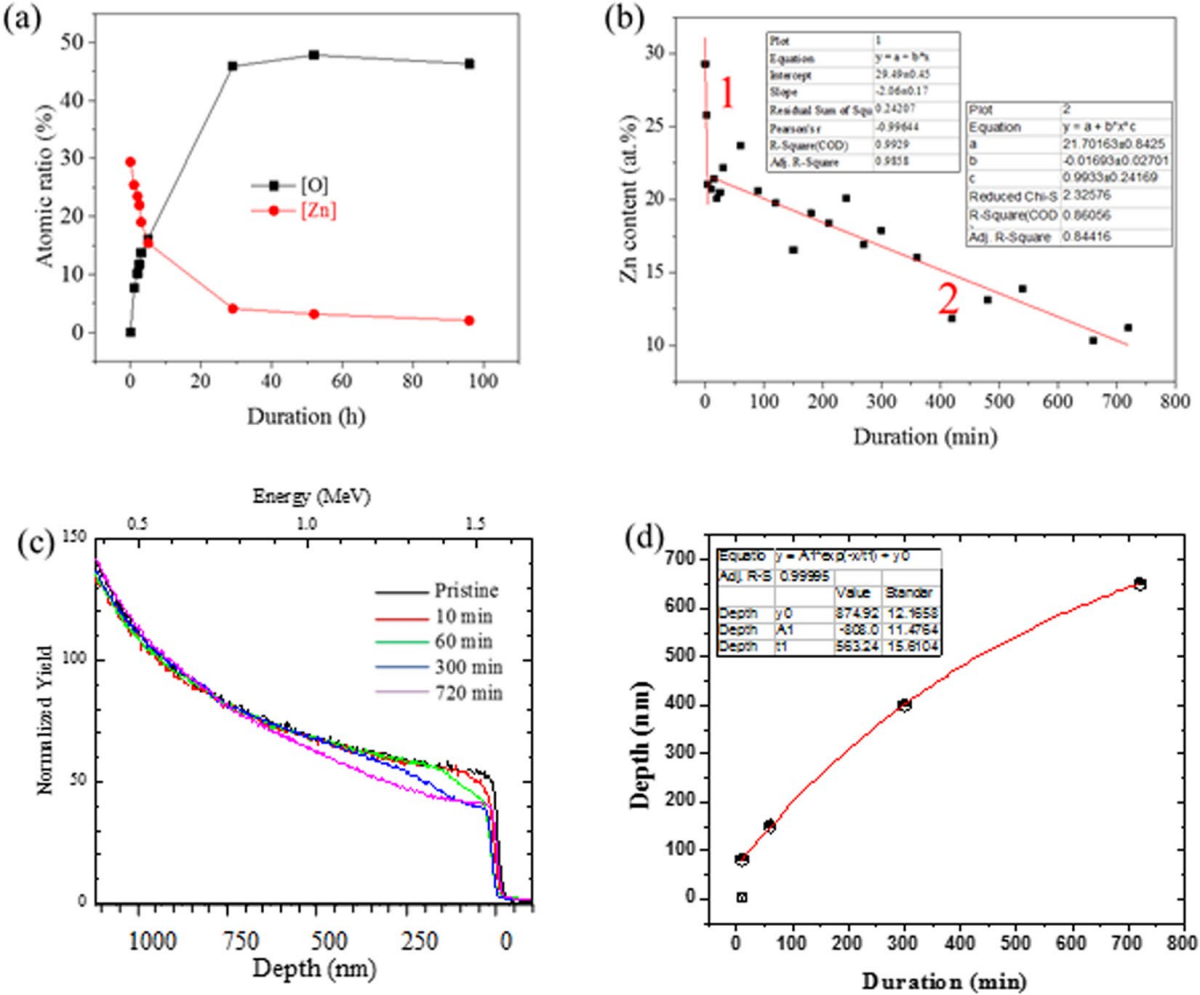
(**a**) The EDS analysis of [O] and [Zn] on the surface of the samples de-alloyde at 20 °C. (**b**) The Zn content of PIXE analysis plotted with duration (min). The range of fitting plot 1 is 0~10 min. The range of fitting plot 2 is 10~720 min. Pristine material CuZn30 dealloyed with 1 M NaOH at room temperature. (**c**) The RBS analysis on dealloyed CuZn30. Dealloyed with 1 M NaOH at room temperature. (**d**) Penetrated depth of oxide layer plotted against the process duration and fitted with polymer equation. The penetrated depth of oxide layer was set at 0.5 μm.

The EDS analysis shown in Supplementary materials S4 (EDS analysis of de-alloyed materials) indicated that the sample had lost approximately one third of Zn content (10 at. %) after dealloying for 3 h (Fig. 2(a)). At the same time, the O content increased with decreasing of Zn content. This indicated that the Cu has partly converted into Cu oxides with dealloying progress. Therefore, the dealloying process of brass by NaOH etching may contain two sub-processes: etching of Zn content and precipitating of Cu content. The EDS analysis can only reveal the variations in surface composition. In the brass system, the Zn atoms would however migrate, following “an infinite cluster” route from the matrix to the surface^17^. Therefore, the variation in surface composition may not be accurately revealing the variation of matrix composition during the process. The EDS analysis revealed variations in surface composition, while the PIXE analysis revealed volume composition changes. PIXE allowed probing a volume of approximately 8 to 10 µm in depth, depending on the exact porosity and density of the medium. Information on alkali dealloyed samples as a function of process duration is presented in Fig. 2(b). The variation in Zn content can be divided into two time periods. First, a dramatic decrease from 29.4 at.% to ~21 at. % within the first 10 min, at a rate of ~1 at.% · min^−1^ was observed. This was followed by a second steady decrease from ~21 at. % at 10 min to ~10 at. % at a much-reduced rate of 0.017 at. % · min^−1^ after 720 min. The slight local variation in composition may be attributed to the inhomogeneity of different *ex-situ* samples used for this analysis.

The samples were also analysed by RBS to evaluate the composition depth profile (Fig. 2(c)). The RBS analysis has a limited depth of analysis which depends on the used He beam energy. In the current system, CuZn30, with a total thickness of 25 μm and a beam energy of 2 MeV only approximately the first micrometer was accessible. Due to the very similar mass of Zn and Cu the depth profiles of the two elements strongly overlap and the Zn/Cu ratio can only be roughly estimated at the very surface of the material. The continous decrease in density on the Cu edge of the data curve around 1.55 MeV indicates the replacement of Cu atoms and very likely the formation of Cu oxide materials^40^. This decrease in density suggested that the oxidation front continuously penetrated into the material as a function of dealloying duration while the degree of oxidation decreased with depth. A pure CuO to Cu interface would exhibit a clear and sharp step profile rather than a slope edge. The slope edge of data curve suggests that the dealloyed surface requires a very long duration to remove all of the Zn and convert the material into pure CuO^40^, supporting the assumption that some Zn could be semi-permanently trapped within the structure due to concentration polarization within the solution or surface passivation upon oxidation. Since the copper oxides are regarded as the mark of Cu corrosion, the penetration depth of the oxides may be directly linked to the frontline of dealloying. Thus, the formation of copper oxides may also directly correlate to the penetration depth of the pores, which was approximately 150 nm after 60 min. The penetration depth of CuO was plotted against the process duration in Fig. 2 (d). The thickness of the CuO layer, function *D*(t), dependent on the duration, was fitted with a first order exponential function, with a R^2^ fitting of 0.99995, yielding:

1 $${D}({t})=874.9-808\cdot {e}^{(-{\rm{t}}/563.24)}$$

where *t* is the process duration (min). Since both sides of the sample have been dealloyed, the thickness *D*(t) is the total thickness of both sides in our experiments. The bevel edge of the RBS Cu/Zn signal shown in Fig. 2 (c) represents a continuous distribution of copper oxides. This edge indicates the presence of a transition area in which the quantity and relative density of the oxides decreased with respect to the penetrating depth^40^. In this area and towards the surface, increasingly larger copper clusters had been etched and precipated as copper oxides until all of the exposed copper atoms had been consumed and a skin of oxides was formed.

In front of the transition area the Zn content had been transferred to the surface and contacted with bulk etching solution. The Zn atoms formed a series of discontinuous vacancies in the frontline of the dealloying process. These vacancies may have transferred into a matrix of the materials due to migration of Zn atoms^17^. These phenomena indicate that the process of dezincification is quicker than the formation of copper oxides. Only 10 at.% (30 at.% in pristine) residual Zn remained after 12 h while the penetration depth was 3 μm on both sides of the metal film, out of a total thickness of 25 μm. The inflection point after 10 min of dezincification indicated the start of a strong concentration polarisation layer within the solution and near the metal surface. The formation of pores is therefore governed by continuous precipitation of metal oxides and the kinetics were different from that of etching Zn metal.

### SAXS analysis of *in-situ* dealloying experiments

The *in-situ* SAXS analysis was performed to evaluate the kinetics of pore formation and assess the effect of temperature on the precipitating process.

**Figure 3:**
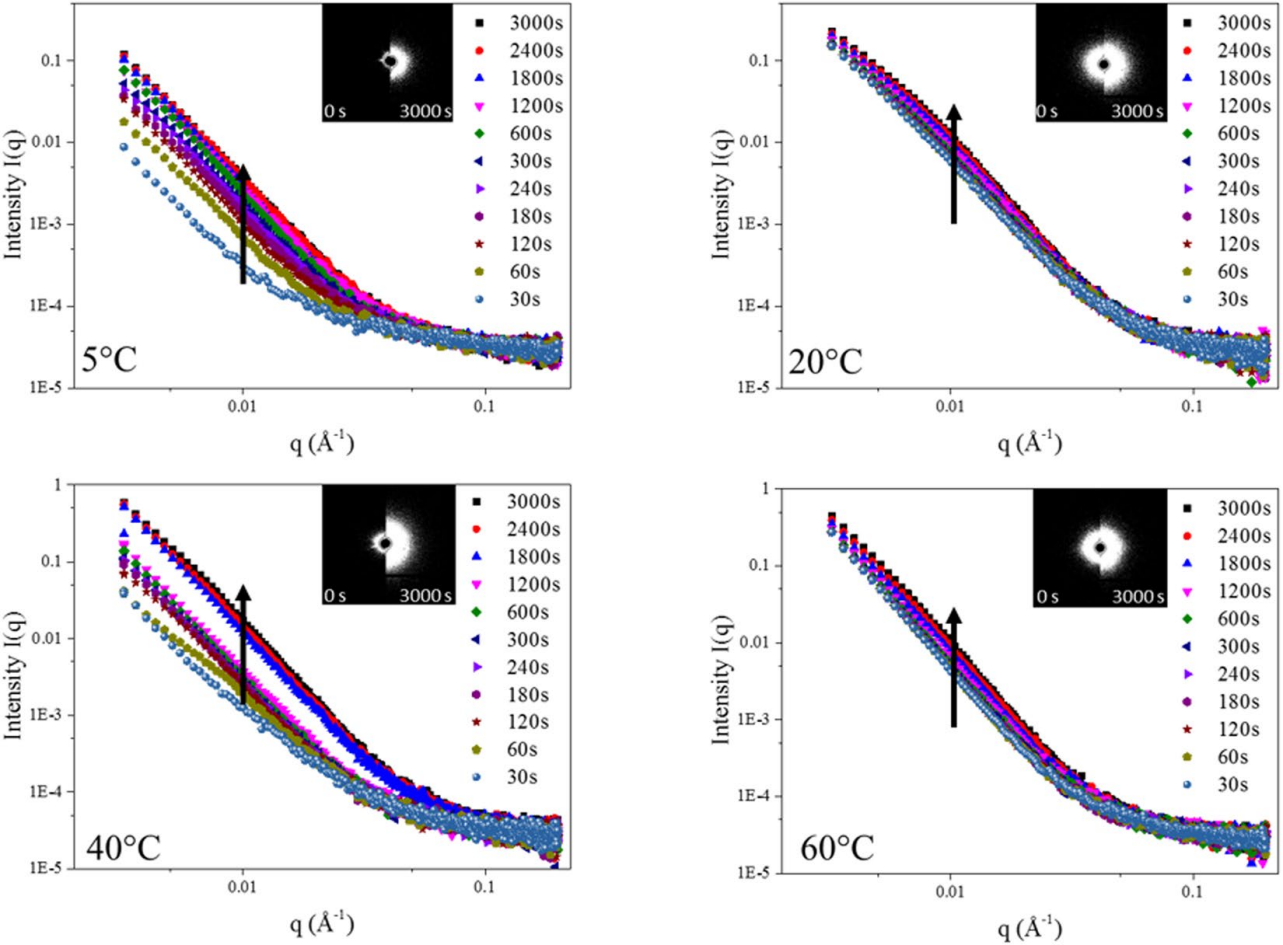
Raw data curves of *in-situ* SAXS dealloying test at 5 °C, 20 °C, 40 °C and 60 °C. The insets show the typical scattering patterns corresponding to the beginning and the end and highlighting the change in the distribution of the scatters. The arrow represents the intensity increase with increasing duration.

The raw integrated scattering profiles, corresponding to the sample of *in-situ* dealloying data, are shown in Fig. 3, while the correlated typical scattering patterns are shown as the insets. The intensity *I*(q,t) notably increased as dealloying progressed. Most of the intensity increase appeared in the low *q* region, *q* < 0.1 Å^−1^, suggesting primarily morphological changes for large features. Besides the intensity increase, with increasing dealloying time a broad scattering knee appeared in a range from 0.003 Å^−1^ to 0.09 Å^−1^. The contrast and overall intensities of these knees were however too weak to precisely locate their width and breadth.

The SEMs of *ex-situ* dealloyed samples for various process durations are shown in Fig. 1 and Figures S14–18 previously discussed, the morphology of the dealloyed Cu-Zn samples offered very low porosity and extremely coarsened ligaments while surface precipitates were found to be more prominent with an increasing etching solution temperature. The ligaments and precipitates were found to become slightly coarsened with an increasing duration while the visible size of dealloyed pores appeared relatively constant. This trend suggests that the rough surface may scatter more beam than the liquid/solid interface across dealloyed pores. As opposed to previously reported dealloyed morphologies based on more noble metals, such as AuAg^41^, the 3D dealloyed Cu-Zn structure does not offer highly regular pore shapes. The *q* range of the corresponding knees therefore arises from the entire size distributions.

Based on the results of RBS and SEM micrographs (Fig. 2(b,c)), the relative thickness of the de-alloyed Cu-Zn system may only reach up to 20% of the sample thickness. Therefore, up to 80% of the sample is not dealloyed and only generates amorphous background signal during X ray scattering experiments. To correct for this, the raw data were normalized by a division method rather than the conventional subtraction method to amplify the intensity of the resulting plot of relative intensity serves to highlight the peak/knee positions but cannot be used for more detailed analysis. This normalisation process is only used to get the q-position of the knee as shown in Supplementary Materials: S5.

**Figure 4:**
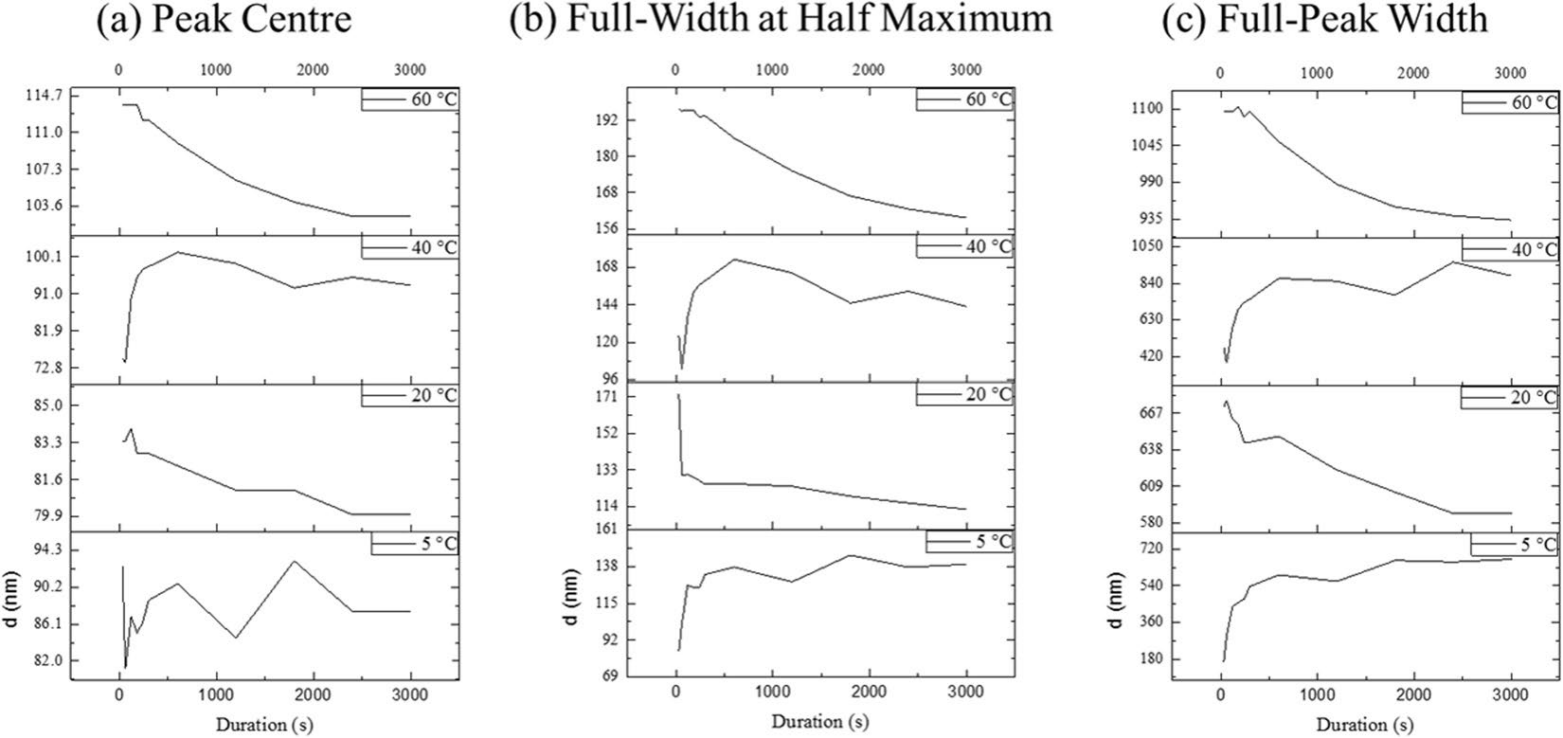
Peak shifting with duration increasing. (**a**) Peak centre (**b**) Full width at half maximum of the peak (**c**) Full-peak width of the peak. Dealloyed of Cu-Zn with a 1 M NaOH solution and for 3000 s; details on the peak fittings are provided in the supplementary information.

The centre of knee distributions was evaluated from these fittings and correlated to the highest frequent distances of neighbouring solid/liquid interfaces. The surface morphology of dealloyed *ex-situ* samples is shown across the SEMs in Figure 4~18 and the ligament was clearly coarsened with duration increasing. The shift of normalized centre distributions for different dealloying procedures is shown in Fig. 4(a). The knee centre position upon dealloying at 5 °C was found to be relatively stable, suggesting little impact of the low temperatures on the pore size distribution. Oscillations of the values around a mean value of 13~15 nm in the physical space were however visible and attributed to the formation of an oxide layer. The centre position of the samples treated at higher temperatures was however found to shift to higher values with dealloying duration increasing. This aspect confirmed that the relative stable scattering peak was correlated to pore size rather than ligament size.

In the first phase, a sharp reduction in size occurred, followed by a second phase in which the mean average size was found to decrease at a much slower pace. This result, in excellent correlation with the PIXE data, suggests a reduction of physical pore size over time, which is consistent with the etching mechanism based on fast solubilization of both Cu and Zn metals. Although Zn is more preferentially etched, likely during the first steps of the dealloying process, Cu will also be slowly solubilized over time and partial dissolution may occur over the scope of minutes to hours. At 20 °C the shift was however found to be extremely limited, with a change of only approximately 4 nm, while at 40 °C and 60 °C, the variations were up to 27 nm and 16 nm, respectively, that were rather significant. Interestingly, the values for the 40 °C samples were found to initially increase significantly prior to dropping back for longer durations. This phenomenon may be due to the etched voids, liked growth period of Au-Ag dealloying process^41^.

The Full-width at half-maximum (FWHM) of the peaks revealed critical information about the size distribution of scattering features (Fig. 4(b)). The FWHM of curves at 5 °C was assessed and found to plateau after a significant increasing in intensity at the early stage of the dealloying process. A similar trend on curves of 5 °C can be found for the full-peak width (FPW) (Fig. 4(c)). The FWHM of the samples dealloyed at 20 °C was found to decrease consistently over time, while at 40 °C the FWHM strongly decreased over the first few minutes of the dealloying process prior to reaching a clear plateau. The FWHM at 60 °C was found to have decreased consistently until the test finished. However, except for the samples at 5 °C, the FPW of curves between 20 °C and 60 °C monotonically decreased with increasing duration. The changes in the width of the distribution may be related to the shape and aspect ratios of the pores^41^. The neighbouring distance of liquid/solid interface of dealloyed Cu-Zn is narrowing with duration increasing, which is interestingly opposite to what happened with the Au-Ag system^41^. This change in behaviour indicates that the dealloyed pores of Cu-Zn tend to be equiaxed and uniform and that precipitation or surface deposition may play a more significant role in the ligament growth and formation compared to more noble metal alloys etching^41^.

**Figure 5:**
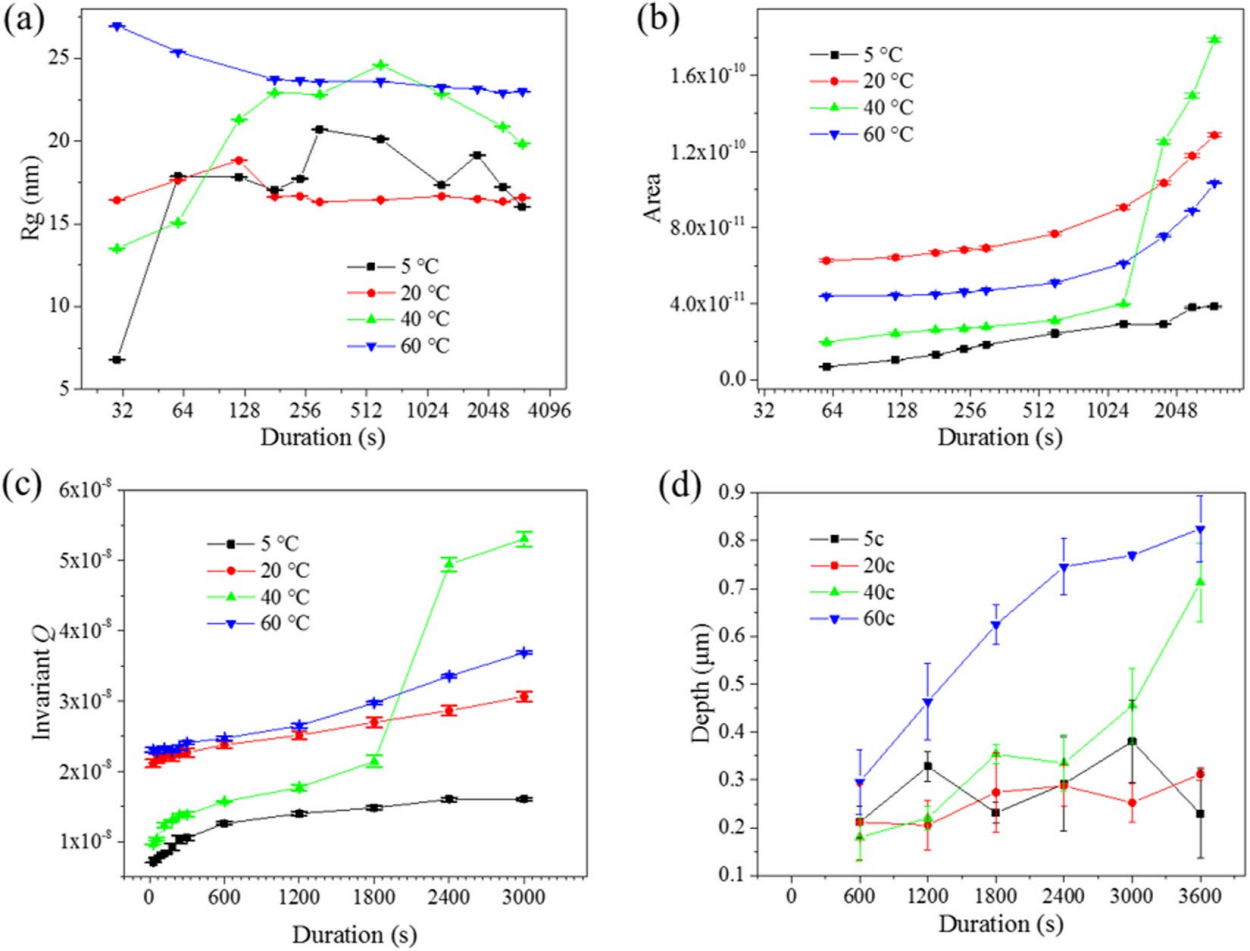
(**a**) The radius of gyration *R*_g_ at different solution temperatures plotted against the process duration. X axis (duration) was plotted with Logarithm binary. (**b**) The surface area A calculated through Porod’s law. (**c**) The Porod invariant *Q* at different temperatures plotted against the process duration. (**d**) The penetration depth of dealloyed region, measured on cross-section view of *ex-situ* dealloyed samples. The value of *A* (40 °C) suddenly doubled at 1800 s. At the end of the *in-situ* tests (3000 s), the sample dealloyed at 40 °C offered the largest surface area, followed by the 20 °C dealloyed samples and the 60 °C dealloyed samples. The surface area of 5 °C dealloyed sample was always the smallest among the 4 series.

The radius of gyration *R*_g_ was also used to estimate the average size of scattering features across dealloyed Cu-Zn samples. Examples of these fits are provided in the Supplementary Information (Figure S11). These fits were performed assuming a diluted system of pores within the metal matrix, suggesting that no scattering features were segregated, as shown for the Guinier fits in the Supplementary Information^42^. The Guinier region of dealloyed Cu-Zn alloy was chosen around the *q* range of the centre of the knee, where the sharpest region of Guinier plot is located. The resulting *R*_g_ values are plotted against the dealloying duration at different temperatures (Fig. 5(a)). The *R*_g_ upon dealloying at 60 °C was found to exhibit the largest value, initialy at 27 nm prior to progressively and slowly decrease to 23 nm by the end of the experiment. The *R*_g_ at 40 °C presented a similar trend except that it quickly increased from 11 nm to 25 nm over the first 600 s prior to slowly reducing to 20 nm, while at 20 °C, the *R*_g_ increased from 16 to18 nm over the first 120 s prior to plateauing around 16 nm. Last, the *R*_g_ at 5 °C was found to be highly unstable, and to fluctuate between 7 and 21 nm for up to 300 s, prior to dropping and stabilizing around 16 nm until the end of the experiment. This result suggests that the pores in dealloyed Cu-Zn samples were shrinking with the duration, which may be related to oxide precipitation and ligament coarsening. The coarsened ligaments have reduced the distance between neighbouting interfaces on dealloyed surface.

The surface area A of the material was obtained after fitting the Porod law across this range of data (Equation 12) and was plotted against the dealloying duration, as shown in Fig. 5(b). The Porod region was chosen for *q* values between 0.05 and 0.1 Å^−1^. This range was selected to satisfy the theoretical requirement that 1/A ≫ *q* ≫ 1/*R*^43^, where A is the surface area of the sample. The initial relative surface area *A* of each sample was *A* (20 °C) > *A* (60 °C) > *A* (40 °C) > *A* (5 °C). After this initial step the surface area was found to increase with respect to increasing dealloying duration up to 1800 s. The increase of surface area is expected as the penetration depth of the pores increases with dealloying progression. The surface area evaluated from the Porod’s Law corresponds to the scattering interfaces of the sample. The step at 40 °C dealloyed sample between 1200 s to 1800 s may be a statistical error caused by the beam probing an area containing large pores, or else offering an initial much larger surface roughness, facilitating pore nucleation. Such large pores can be found randomly on the SEM images of the *ex-situ* dealloyed samples and are likely caused by the rolling process used to form the films (Figure S16).

The Porod invariants, *Q*, is used to correlate the scattered beam energy and volume of material *V*_0_, from which the main X-ray beam was scattered^44^. The invariant of the *in-situ* dealloyed Cu-Zn samples was plotted against the dealloying process duration, as shown in Fig. 5(c), while the penetration depth of the dealloyed pores in *ex-situ* dealloyed samples are presented in Fig. 5(d). The penetration depth of the pores was found to increase with respect to increasing process duration, and to increasing solution temperature (from 20 °C to 60 °C). The penetration depth of the 5 °C dealloyed samples was found to pleateau similar to the observation for *R*_g_ which was attributed to the stabilization of the dealloying process upon reaching a critical depth.

**Figure 6:**
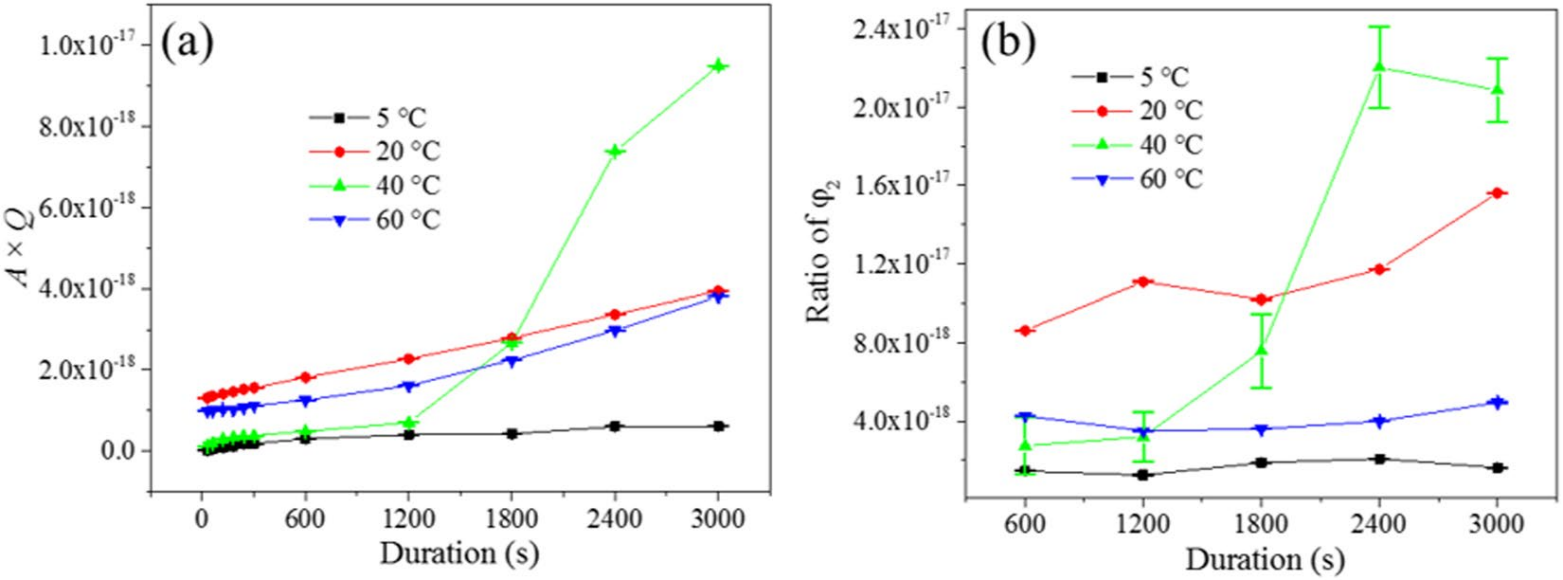
(**a**) Product of A*Q plotted against the process duration. (**b**) Calculated solid phase fragment φ_2_ plotted against the process duration.

The product of invariant *Q* and surface area *A* was plotted against the process duration as shown in Fig. 6(a). In the Cu-Zn system, the penetration depth of the dealloyed region increases and the residual Zn in the dealloyed region decreases as the process progresses. Therefore, the penetration depth *T*_p_ of dealloyed region was measured from cross-section views of *ex-situ* dealloyed samples to yield the physical scattering volume *V*_s_ (Equation 5). According to Equation 11, A*Q divided by *V*_s_ is proportional to the solid phase (ligaments) ratio *φ*_2_. The solid phase (ligaments) ratio *φ*_2_ plotted against the process duration was presented in Fig. 6(b). The *φ*_2_ in the Cu-Zn system does not represent the progress of the dealloying process. However, φ_2_ is related to the formation of dealloyed structure. The ratio φ_2_ increased with process progressing at the room temperature (20 °C), while at 5 °C and 60 °C it remained relatively stable. At 40 °C, the *φ*_2_ has increased extremely fast between 1200 s to 2400 s prior to plateauing, or even decreasing slightly.

For all experiments above 5 °C, the average size of the scattered features at any temperature and their associated distribution decreased with de-alloying process duration prior to reaching a plateau which is likely related to the precipitating rate of dissolved Cu (Supplementary Material S6). Therefore, the centre of the scattering knee shifted to a smaller average pore size due to the increase in ligament volume of the material. This shift of the scattering knees across the Cu-Zn pattern was much smaller than that previously reported in similar conditions on Au-Ag system^41^. These phenomena suggested that the *in-situ* de-alloyed CuZn samples might be still in the incubation period or early of transformation period even after 3000 s.

The de-alloying process is therefore found to alternate between etching and precipitation mechanisms to proceed. Indeed, the precipitation mechanism is concentration dependant, and will therefore likely only kick-in when sufficient building materials are available within the pores or across the surface. Likely, this solid-liquid transfer mechanism generates pulsed mechanisms, whereby concentration polarization led to ions being transferred from the pores to the bulk liquid, and to oscillations of the concentrations therefore radially to the surface. This aspect explains why the kinetics of de-alloying appeared to decrease much faster for the CuZn alloys compared to the AuAg alloys. The pore penetration was hindered due to the contra-diffusion and precipitation happening simultaneously. The coarsened ligament of 60 °C *ex-situ* de-alloyed sample (Figure S17) and stable ligament phase ratio *φ*_2_ of 60 °C *in-situ* de-alloyed sample (Fig. 6(b)) suggested that the etching and precipitating may therefore coexist on the probed volume of sample during the *in-situ* SAXS experiment.

## Conclusions

In summary, a chemical dealloying process on Cu-Zn alloy is a combination of competitive etching and Cu precipitating processes. The combined RBS, PIXE, SEM and SAXS data helped understand both the pore formation and the kinetics of dealloying of the Cu-Zn system. The precipitating sub-process was primarily associated with the precipitation of dissolved Cu atoms, into CuO in the present case. The morphology of de-alloyed porous framework and the thickness of the porous layer were mainly correlated to the precipitating process. Specifically, with temperature increasing, the morphology of the porous frameworks became coarsened significantly.

This temperature assisted process allowed the generation of layer-by-layer de-alloyed sheets which thickness could be controlled down to less than a few hundreds of nanometres, therefore opening new manufacturing opportunities in the fabrication of ultra-thin porous films or porous framework with multiple layers.

## Electronic supplementary material

Supplementary information

## Data Availability

The date of the work are available upon contacting the corresponding authors of the manuscript.
