# Supplementary material for: An in-situ small angle x ray scattering analysis of nanopore formation during thermally induced chemical dealloying of brass thin foils
Source: Sci Rep. 2018 Oct 18;8:15419. doi: 10.1038/s41598-018-33787-z (PMC6193925; doi:10.1038/s41598-018-33787-z)
Supplement: Supplementary file 1 — Supplementary information [file 41598_2018_33787_MOESM1_ESM.pdf]

Supplementary materials

Title

An in-situ small angle x ray scattering analysis of nanopore formation during thermally induced chemical dealloying of brass thin foils

Authors

Bao Lin[1], Max Doebeli[2], Stephen Mudie[3], Adrian Hawley[3], Peter Hodgson[1], Lingxue Kong[1], Ralph Spolenak[2], Ludovic F. Dumée[1#]

Affiliations

1 Deakin University, Institute for Frontier Materials, Waurn Ponds 3216, Victoria, Australia

2 ETH Zurich, Vladimir-Prelog-Weg 5, 8093 Zürich, Switzerland

3 Australian Synchrotron, Clayton 3168, Victoria, Australia

#Corresponding author: ludovic.dumee@deakin.edu.au; +61410131312

Supplementary Information

S1. Pristine material characterization

The pristine foils were shown in Figure S1. It is a face-centre cubic (FCC) single phase alloy sheet, which lattice constant was around 3.69 Å. The pristine sheet was fabricated by hot rolling process, and the average grain size is approximate 2 μm. In addition, the surface roughness of pristine sample is 21.9 nm.

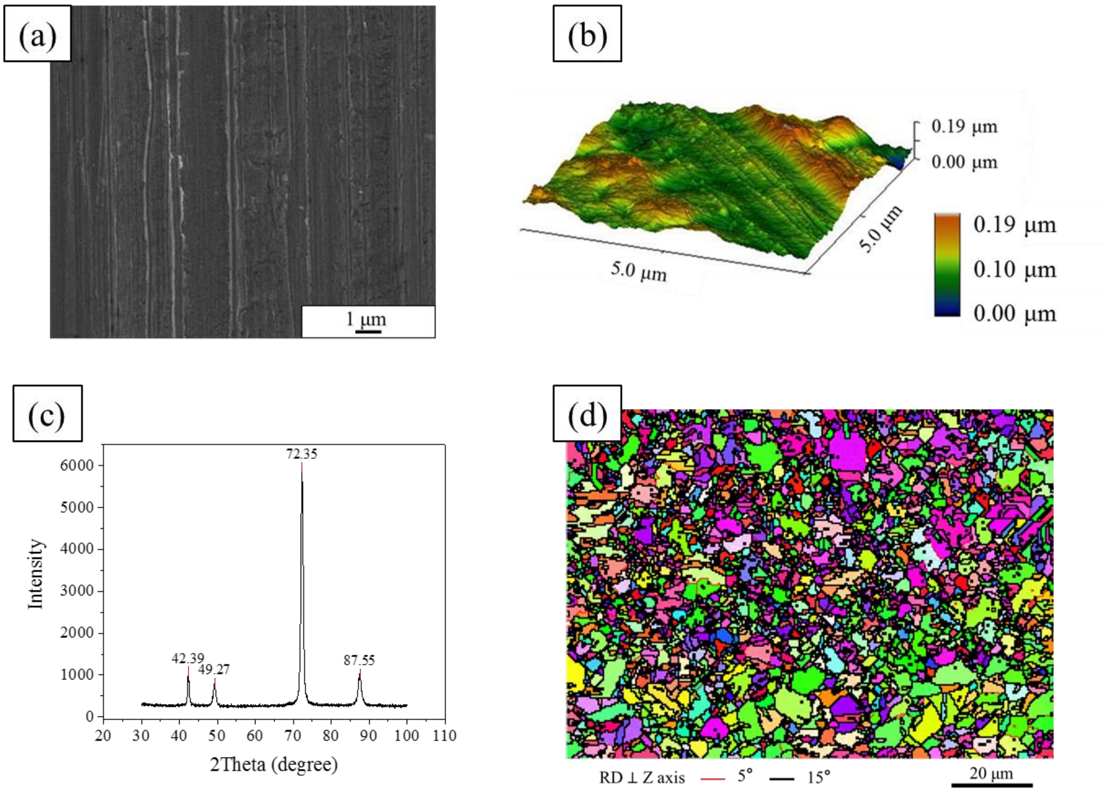

Figure S1 (a) SEM of pristine CuZn30. (b) AFM mapping of pristine CuZn30. (c) XRD pattern of pristine CuZn30. (d) EBSD mapping of pristine CuZn30

Electron Back-Scatter Diffraction (EBSD) was used to generate orientation maps of grains, performed with a Nordlys S (Oxford (HKL), UK) across the pristine metal leaves. The orientation analysis performed with the Nordlys S EBSD detector (HKL) at 20 keV in high current mode and analysed with the Aztec Software (Oxford, UK). The scanning area is 200 μm × 150 μm while the step size was 0.4 μm. The rest of the parameters for EBSD scanning have not been set and default manufacturer values were used. The original EBSD data is an orientation map which reveals directly the size, shape and orientation of grains. Based on the

orientation map, Misorientation Angle Distributions (MAD) could be obtained according to the statistics of the orientation map. Misorientation angle referred to the inter-angle of two grains which can reflect the manufacture process of pristine materials or deformation occurred during operation or modification. The misorientation angle of neighbouring grains correlates to the mismatch of atoms on the grain boundaries which was previously shown to correlates to the electrochemical properties, and particularly here to the anti-corrosion properties of materials [1]. The Pole Figure (PF) and Inverse Pole Figure (IPF) are stereographic projections of normal direction of each grain. The standard stereographic projection please refer to the literature [2]. These Figures of stereographic projection were used to visualize certain types of fibre textures.

S2. Cell preparation for the dealloying process

A schematic of the cell used in this study is presented in Figure S2.

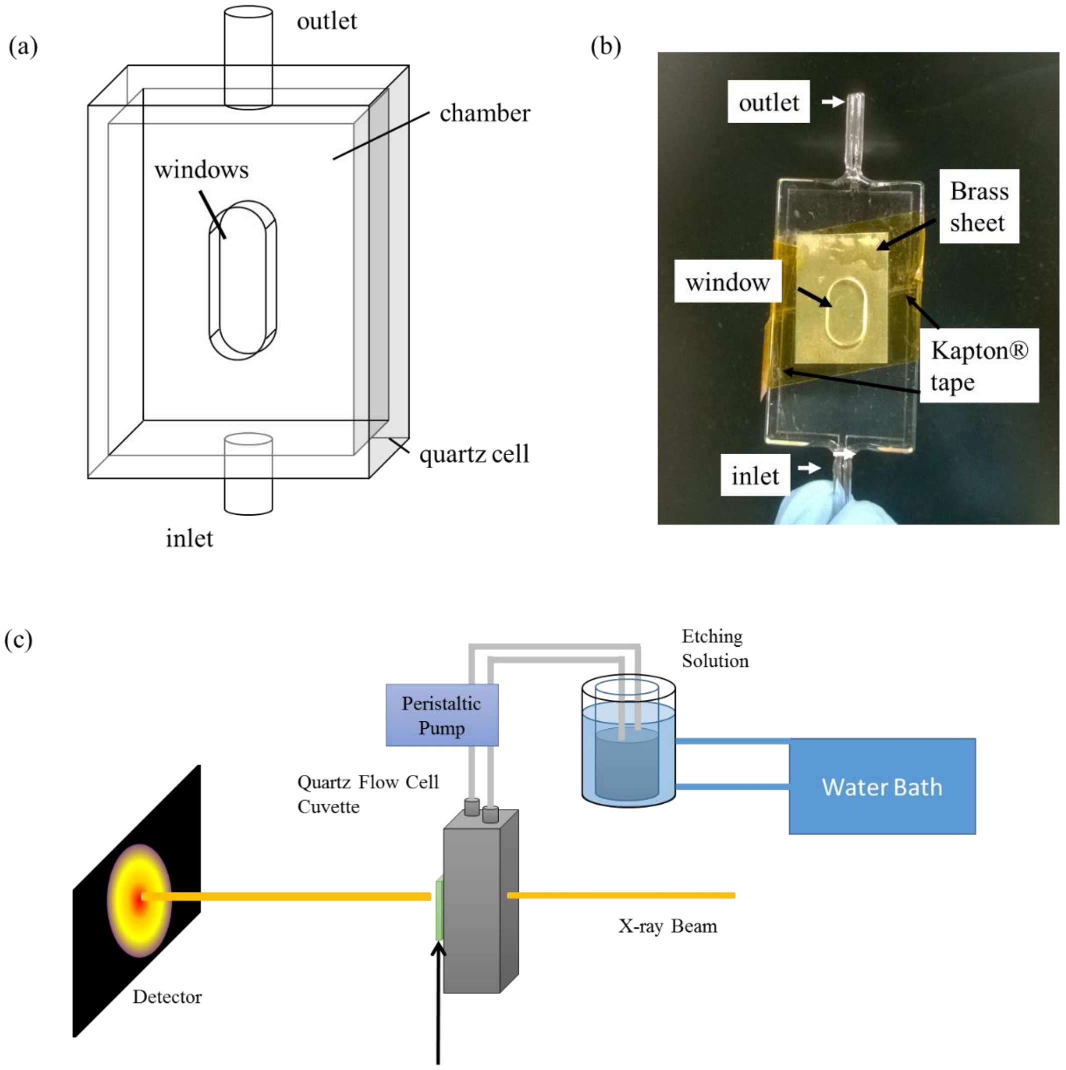

Figure S2 (a) Schematic of the flow cell. (b) Picture of quartz flow rig. (c) Schematic of in-situ experiment on SAXS. The etching solution was stored in a jacketed beaker, which is connected to a water bath for temperature control, and pumped with a peristaltic pump with a flow rate of 30 mL·min^{-1}. The working surface of sample is facing the incident direction of beam.

S3. SAXS modelling and normalization

The illustration of in-situ dealloying test was shown in Figure S2 (c). The thickness of the porous layer (T_P) shall increase with dealloying duration increasing, while the thickness of dense material (T_S) shall decrease simultaneously upon material removal. However, the thickness of dealloyed porous sample is smaller than that of the pristine sample [3], suggesting that the thickness of the whole sample is decreasing with duration increasing. The volume shrinkage at very early stage of dealloying process is however difficult to measure experimentally and thus too small to affect the beam. In this case, the whole thickness of the sample was therefore assumed to be constant during the test, which is $T_P(t) + T_S(t) = T_P(0) + T_S(0)$. Last, the thickness of the liquid layer (T_l) across the cell was also constant.

The liquid layer was filled with the etching solution and ions generated from the dealloying process. The ions in the solution are changing during the experiment. Besides the consumption of etchant ions, new ions were always generated with dealloying process progressing. The etching solution in this experiment was continuously recirculated from a solution reservoir to the flow cell. This water flow is likely that adding a dynamic background to the changing sample. As discussion previously, the impact of the solution concentration change over time was too small to be distinguishable by the SAXS detector. The etching solution was therefore regarded as a constant background.

The typical dealloying porous structure was therefore considered as a binary phase system, composed of solid metal and liquid solution, composed of metal ions, hydroxides and etchant molecules. The first component therefore corresponds to the ligaments while the other one represents the pores across the materials. Although the presence of the ions in the solutions, as well as the density of the liquid, being > 90% water, affected the intensity of the background, the direct presence of metal ions, complexes or hydroxides was not found to affect at all the patterns, primarily due to their low concentrations (less than 0.0002 M after dealloying) but also due to their size, to small to be detectable on the utilized camera length [4].

S2.1 Phase contrast determination

Since one cannot directly distinguish the two phases from other with the SAXS data, this binary phase system was treated as a diluted particle-solution system [5]. The Porod invariant Q was introduced to correlate the scattered beam energy and volume of material V_0, which was scattered from the main x-ray beam [6], as shown in Equation 1.

$$Q = \int_0^\infty q^2 I(q) dq = 2\pi^2 V_0 (\Delta\rho^2) \qquad \text{Equation 1}$$

Where, $(\Delta\rho^2) = (\rho_1-\rho_2)^2\varphi_1\varphi_2$ is the contrast in binary system [6]. The ρ_1 and ρ_2 correspond to the density of both phase 1 and 2 respectively, while φ_1 and φ_2 represent the volume ratio of each phase. In other words, the beam scattering happened at the interface of the different phases. In a typical ex-situ experiment, the vacancy of porous material was filled with air [7]. So, the ρ_2 is the density of air while φ_2 is the porosity of material.

In in-situ dealloying process, the pore formation was regarded as the formation of a 2nd phase. However, the cavities and pores formation was due to the dissolution of one component from the alloy, which was primarily Zn in this work. The dissolution process, therefore leads to the formation of a core-shell structure, where the core of the material ligaments is still brass, while the outer shells of the ligaments is primarily Cu and Cu oxides. This phenomenon will expand during the penetration across the materials and a gradient of oxide formation may be generated in case of metal oxide precipitation. The final material will have at least three different phases concurrently present across the matter over time. A schematic of different phases is shown in Figure S3.

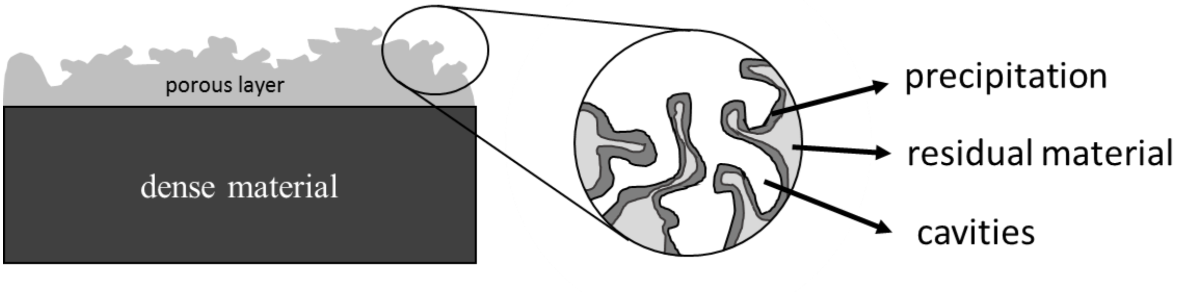

Figure S3 Schematic of phase composition of dealloyed brass

Furthermore, the pores and cavities were filled with the etching solution and metal ions. The metal ions which were from the etching of alloy may vary during the experiment. In other words, the density of the liquid phase is changing during the experiment. This variation could not be accurately evaluated by linear or one-order linear differential equation. The contrast $(\Delta\rho^2)$ was therefore modified in order to satisfy trinary phase system (Equation 2).

$$(\Delta\rho^2) = (\rho_1 - \rho_2)^2\varphi_1\varphi_2 + (\rho_1 - \rho_3)^2\varphi_1\varphi_3 + (\rho_3 - \rho_2)^2\varphi_3\varphi_2 \qquad \text{Equation 2}$$

Where the ρ_1 and φ_1 represent the density and volume ratio of pristine brass phase respectively; while the ρ_2 and φ_2 represent the vacancy phase which is filled by solution and the ρ_3 and φ_3 correspond to the ligament phase which is primarily copper / copper oxide. A number of assumptions were put in place to simplify the data analysis, as discussed in following section.

S2.2 SAXS intensity normalisation

The intensity of the raw data is the sum of the scattering intensities and background intensities in the system. Thus, the background data were subtracted from the background (blank reference) to yield the scattering data. In this in-situ dealloying process on the CuZn30 alloys, the porous part of the material upon dealloying represents a very small volume compared to the dense part of the base foil. Therefore, the initial condition of each dealloying system, including the pristine material and the etching solution, were regarded as the general background and each sample was its own reference. The raw data was normalized with background data by dividing it [8] to enhance the main knee position (Equation 3).

$$\mathrm{I_{scattering}} + \mathrm{I_{background}} \Rightarrow \frac{\mathrm{I_{scattering}}}{\mathrm{I_{background}}} + 1 \Rightarrow \frac{\mathrm{I_{scattering}}}{\mathrm{I_{background}}} > 1 \qquad \text{Equation 3}$$

The intensity I(q) was converted into a relative intensity ratio I'(q) = I(q) / I_0 (q), in which I′ (q)-1 ≥ 0. The knee range can be easily located with the normalized curves and their intensity compared upon increased duration. These normalized curves are however not suitable for Guinier analysis since the relative intensity has changed the 1st differential curve of raw SAXS pattern [5], while normalization with background subtraction will not change the 1st differential curve.

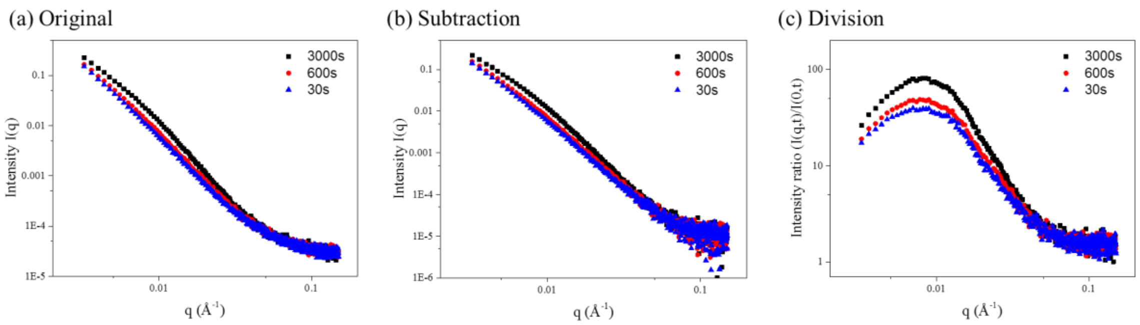

Figure S4 Schematic illustration of a comparison between two normalization methods. (a) Original curves. (b) Normalized curves with subtraction, in which each data curves subtracted the background curves. (C) Normalized curves with division, in which each data curves divided by the background curves

Since most of the peak were not fully visible across in the SAXS q range, an extrapolation method was used to fit and simulate the curve and evaluate the full scope of the knees. Before fitting the knees, the baseline for each curve needed to be subtracted. According to the Equation 3, $I'(q) = I(q)/I_b(q) + 1$, the background intensity $I_b(q)$ is a constant. The whole thickness T_p+T_s was assumed to be constant in above, but the decrease of T_s for long times led to a slight increase of the $I_{background}$ since the density of sample had decreased. Thus, the Equation 3 was modified to take into account the background variation as a function of the dealloying duration (Equation 4)[8].

$$I_{scattering}(t) + I_{background}(t) \Rightarrow \frac{I_{scattering}(t)}{I_{background}(0)} + \frac{I_{background}(t)}{I_{background}(0)} \qquad \text{Equation 4}$$

So $I'_b(t) = I_{background}(t) / I_{backgound}(0)$ and $I(q,t) = I_{scattering}(t) / I\ I_{backgound}(0)$. When t=0, the $I'_b(t) = 1$ and $I(q,t) – I'_b(t) = I'(q,t) \geq 0$. The $I'_b(t)$ at different duration was achieved by linear fitting the I(q,t) with slope = 0, while q value is in an interval from 0.07 $Å^{-1}$ to 0.1 $Å^{-1}$ which was regarded as a constant background area of the signal.

Then, $I'(q,t)$ was plotted against $\log_{10}(q)$ and the curve was fitted with a Gaussian function, which is the normal distribution function. Using $\log_{10}(q)$ instead of q allows for inhomogeneous data density distribution evaluation across the whole q range. The fitting process was performed through the following steps:

1. Plot relative I'(q) versus logarithmic scattering vector q;
2. Locate the highest q value of end of peaks among all curves in one temperature series;
3. Select data picking range from the first q = 0.0332 to highest q value which confirmed in last steps;
4. Fitting selected data with Guassian peak fitting function with nonlinear fitting function of Origin 2015 (academic version, supplied by Deakin University);
5. Extended curve to $\log_{10}$ q = - 4.

The calculation of the Q invariant for the following analysis required the data to not be normalized by division by the $I_{background}$. For that reason, that the background intensity $I_0(q)$ needed to be also extrapolated. If no knee appeared upon normalizing the curves with the background then a Boltzmann function fit, extended to q = 1e^{-4} Å^{-1} was applied.

S2.3 Scattering volume and thickness of the porous layer

The scattering volume V_s was calculated through the Porod invariant Q (Equation 1), where the beam spot area S_B was assumed to be constant (surface of 15,000 µm^2). Therefore, the thickness of porous part (pores penetration depth) T_p was evaluated as a function of scattering volume V_s (Equation 5), where the V_0 is the probed volume of material, φ_s is the volume fraction of porous material.

$$T_p = \frac{V_s}{S_B} = \frac{V_0 \varphi_s}{S_B} \qquad \text{Equation 5}$$

The invariant Q can be calculated by integrating the area corresponding to the knee on the Kratky plot. The Kratky plot was obtained by plotting the scattering vector q versus the scattering intensity I(q) times q^2 instead of I(q) (Equation 6).

$$Q = \int_0^{0.2} q^2 I(q) dq \qquad \text{Equation 6}$$

Then,

$$Q(t) = \int_0^{0.2} q^2 I(q,t) dq \qquad \text{Equation 7}$$

In Equation 7, t corresponds to the duration. At t = 0, then I(q,t) = I_0(q), which yields the background invariant Q_0. The porous layer invariant Q_P(t) is therefore calculated by the difference between Q(t) and Q_0 (Equation 8).

$$Q_P(t) = Q(T) - Q_0 = \int_0^{0.2} q^2\,[I(q,t) - I_0(q)\,]dq \qquad \text{Equation 8}$$

Since only the porous part will scatter in the given q range observed the difference value between I(q,t) – I_0(q) is theoretically nil, except in the peak area. In other words, only the area under the knee is relevant and may be integrated, to further simply the system, from 0 to 0.2 $Å^{-1}$.

$$Q_P(t) = \int_{Peak\ area} q^2\,[I(q,t) - I_0(q)\,]dq \qquad \text{Equation 9}$$

It was necessary to use the extrapolated data to obtain the whole knee area. However, the normalized data could not directly be applied into Equation 9 and a reverse fitting process was applied to the normalized date. Specifically, I"(q,t) was defined as, I"(q,t) = (I'(q,t)+I'b(t)) * I_0(q). Since the value interval of original I_0(q) is ranging from 0.003 $Å^{-1}$ to 0.2 $Å^{-1}$, the I_0'(q) was obtained by extrapolating I_0(q) with the Boltzmann equation, which yielded Equation 10.

$$Q_P(t) = \int_{Peak\ start}^{Peak\ end} q^2\,[I''(q,t) - I_0{}'\,(q)\,]dq \qquad \text{Equation 10}$$

The Q_P(t) is regarded as the scattering energy on the dealloyed part of the sample if assuming the background is constant (assumption still valid in this case, largely within experimental error). Therefore, the scattering volume of the dealloyed part was obtained by inputing the Q_P(t) into Equation 1. Regarding the dense part of the sample, or the non-edalloyed part, which is still part of the background signal, the contrast $(\Delta\rho)^2$ can be simplified into a simple binary system, which is $(\Delta\rho^2) = (\rho_1\text{-}\rho_2)^2\varphi_1\varphi_2$.

The scattering interface generated during the process was generated by the de-alloyed probed material. Thus, the liquid phase fraction φ_1, which is a function of the de-alloying duration in the in-situ de-alloying tests, $\varphi_1(t)$, was regarded as the porosity of de-alloyed sample. In addition, the probed volume V_0 of the sample corresponds to the beam size

multiplied by the thickness of the dealloyed part of the sample. So, the scattering volume V_s = $V_0\ \varphi_1(t)$.

Therefore,

$$SQ \sim V_s\ (t)\varphi_2(t) \lim_{q\to\infty}(q^4 I(q))$$

where,

$$\lim_{q\to\infty}(q^4 I(q)) \ \to constant$$

Then,

$$AQ \sim V_s(t)[1-\varphi_1(t)] \qquad \text{Equation 11}$$

The solid phase ratio φ_2 can therefore be achieved if the Vs(t) was measured.

S2.4 Porod's law and surface area

The Porod's law was used to estimate the relative surface area of sphere particle with a smooth surface [5,9]. In the Porod's law, the intensity I(q) is proportional to the scattering vector q in high q, while the relative surface area S is the coefficient (Equation 12).

$$I(q) \sim S\ q^{-4} \qquad \text{Equation 12}$$

In Equation 11, the exponent '-4' can be written as a generalized form '-α' (Equation 13). The value of α depends on the shape, aspect ratio and roughness of the scattering features. Specifically, the '-α' is may vary between -3 to -4 for rough surfaces, while between -2 and -3 it largely correlates to thin disk, and around -1 it may be correlated to a rod-like geometry. Large Porod exponents are typically considered to represent fractal surfaces, or in other words ordered and patterned thin layers, while small Porod exponents are attributed to volume fractals, such as expanded 3D networks.

$$I(q) \sim S\ q^{-\alpha} \qquad \text{Equation 13}$$

The interval of validity of the Porod law should in theory be smaller than 0.1 Å^{-1} since the scattering features in this range would be attributed to interfaces rather than atoms or bonds. In this project, the Porod's law was used to reveal the surface area of the ligaments. Therefore, the scattering vector interval of interest was taken for the Porod law outside the range of the main knee area, and close to 0.1 Å^{-1}.

S2.5 Radius of Gyration and Average pore size

The Guinier analysis refers to the analysis of the SAXS scattering curve at very small scattering angles. This analysis allows for the direct estimation of two SAXS invariants, the radius-of-gyration, Rg, and the extrapolated intensity at zero scattering angles, I(0).

$$\ln I(q) = \ln I(0) - \frac{{R_g}^2}{3} \cdot q^2 \qquad \text{Equation 14}$$

where the R_g in Radius of Gyration, the q is scattering vector and I is the scattering intensity which is the function of q. The $q \cdot R_g$ should typically be smaller than 1.3 according to the most common definition of R_g [5,10]. Since I(0) is constant, the can be simplified and converted into Equation 15.

$$\ln I(q) \sim -\frac{{R_g}^2}{3} \cdot q^2 \qquad \text{Equation 15}$$

The data was plotted by ln I(q) versus q square, as Guinier plot. The slope of the Guinier plot in Guinier region is considered as the R_g (Radius of Gyration).

S4. EDS analysis of de-alloyed materials

Previous researches typically refer to the residual Zn to represent the progress of dealloying process [11-14]. The residual Zn content is therefore used to correlate to variations in dealloyed morphology. However, the residual Zn cannot be directly revealed the progress of the dealloying since the dissolution of Zn depends whether it has been directly exposed to the etching solution. On one hand, Zn atoms surrounded by Cu atoms are required to be transferred to the surface through Kirkendall effects if they are not directly exposed to the etching solution. The transfer of Zn atoms may be terminated upon passivation of the surface or from a lack of vacancies providing diffusion pathways [15].

Copper oxides are a brass corrosion product formed in a basic solution. The copper oxides are an indication that the Cu metal participated in the dealloying process and was etched by the etching solution. The precipitation of Cu was regarded as an independent process at the end of the last section, which may significantly impact on the dealloyed porous morphology. Therefore, the copper oxides are more relevant to the progress of dealloying process and the morphology of the products.

The temperature was shown to affect the morphology of dealloyed sample due to the change in precipitation rates achieved at different temperatures, as mentioned in the last section. Thus, the [O] composition will theoretically change if the etching solution temperature of the dealloying process is changed. As shown in Figure S5, the variation in [Zn] is going in the opposite way to the variation of [O], in which the absolute atomic ratio (Figure S5) is the average value of the EDS spectrum analysis. Between 5 °C and 40 °C, higher solution temperatures led to lower residual Zn and more Cu oxides formation. However, at 60 °C, the [Zn] increased back up 27 at. % (29.4 at. % for the pristine) while the composition of [O] was set at 8 at. %. The dealloying rate had therefore slowed down when solution temperature was 60 °C. Assuming the precipitated product of Cu is CuO, there is only approximately 4 at. % of Cu belongs to the CuO. The atomic ratio of residual [Cu] and [Zn] is approximately 43 % which is close to that of the pristine material. This trend indicates that the surface of the 60 °C dealloyed sample was composed of precipitated CuO recovering the pristine matrix.

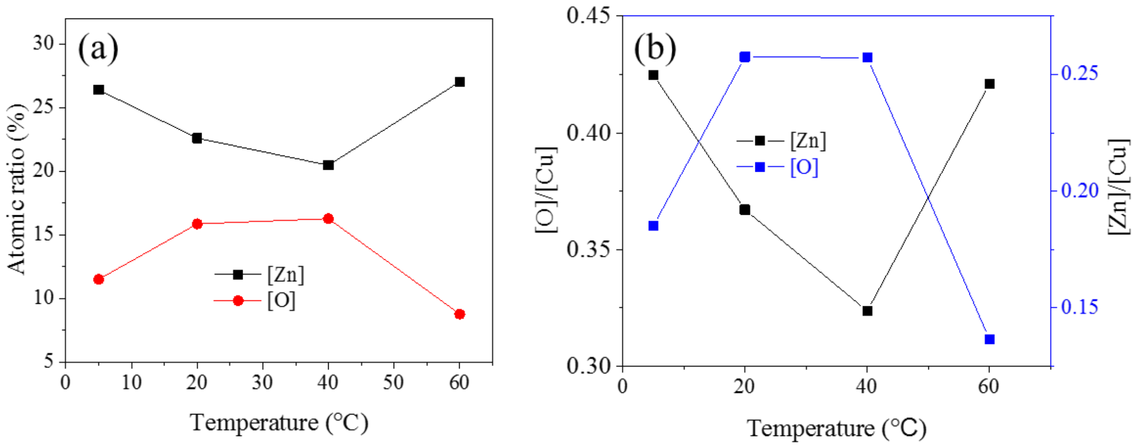

Figure S5 The EDS analysis result of [O] and [Zn] on dealloyed sample's surface fabricated at different solution temperature. (a) Absolute atomic ratio. (b) Relative ratio to Cu. The sample dealloyed with 1 M NaOH for 1 h. Error bar included but extremely small, and difficult to distinguish from the main pattern

The surface composition of a series of dealloyed samples put in contact with the etching solutions for different durations was analysed with EDS and the result are presented in Figure S6 (a). The [Zn] composition decrease with duration increasing while the [O] was increasing with duration increasing. The rate of [Zn] etching was faster initially for shorter times prior to slowing down with process progressing. It almost reached a plateau (~4.3 at. %) after 96 h of duration. The [O] increased significantly with duration increasing and reached a plateau after 29 h of duration. The plateau of [O]/[Cu] is around 0.9 (Figure S6 (b)). This value demonstrates that the precipitation of Cu is CuO, and the residual Zn was in the pristine matrix yet to be contacted with an etching solution. The EDS spectrum and mapping of the 51 h dealloyed sample, shown in Figure S6, demonstrate that the dealloyed surface was covered by a homogenous layer of copper oxide. After 96 h of duration, both [O] and [Zn] however decreased. The reason for this change may be that, first, the fragile CuO porous layer may have been peeled off during the process, while second, the porous layers was progressively converted into a porous framwork with finer ligament due to metal surface atoms recombination, offering less interaction volume for X-ray excitation. In addition, the Cu_2O was perhaps generated or converted from CuO since the pH of solution had decreased in the viscinity of the surface from the strong concentration polarisation induced by the dealloying

process. It is obvisouly noteworthy that the valences of both Cu (Cu(I) or Cu(II)) are not distinguishable by EDS analysis, which only report atomic Cu species as a whole.

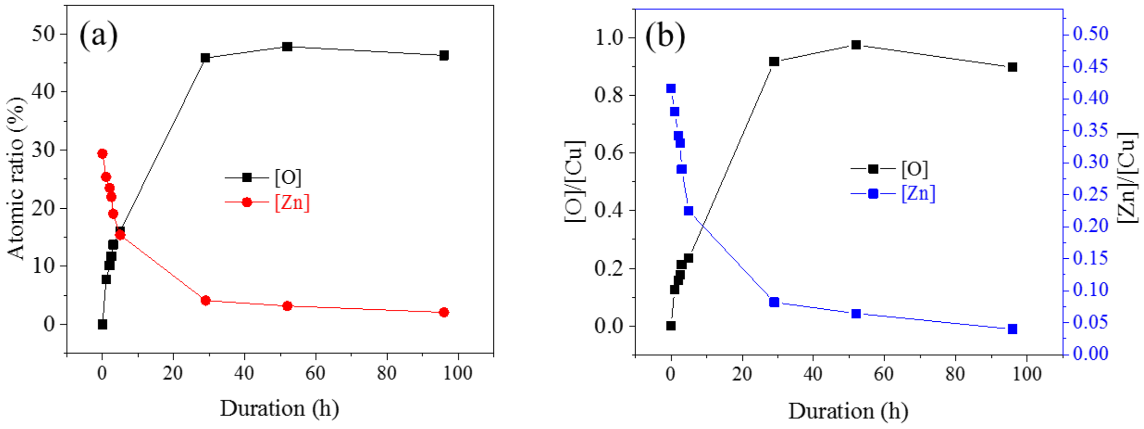

Figure S6 The EDS analysis result of [O] and [Zn] on dealloyed surface of the samples variations with duration increasing (Figure 1 (a)). (a) Absolute atomic ratio. (b) Relative ratio to Cu. The sample dealloyed with 1 M NaOH at room temperature. From SEM analysis

S5. The normalization of SAXS pattern

The contrast and overall intensity of the scattering knees, shown in the Figure 5, were too weak to precisely locate their width and breadth. The main two reasons for this low intensity are that the relative volume of the porous layer, which was generated by the dealloying process and corresponds to the scattering features, is very small compared to that of the overall dense pristine material, while the distribution of these features was likely large at the nanoscale, ranging, as seen across the SEMs in Figure S7, from 20 nm to over 1 μm. These issues therefore limited the volume ratios of the beam scattered by the pore/ligament feature composing the porous layer.

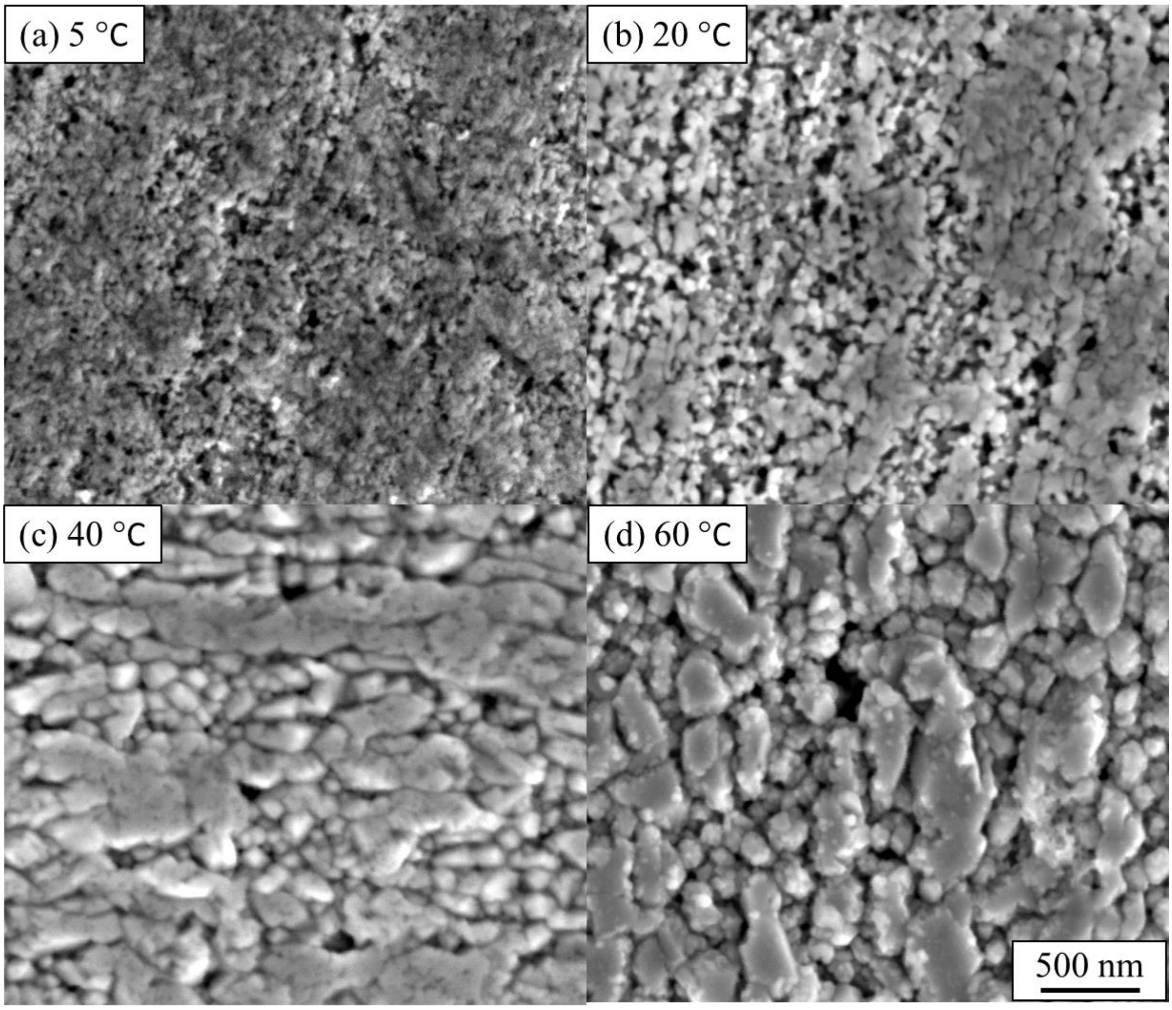

Figure S7 SEMs of dealloyed CuZn30 after in-situ SAXS dealloying tests. (a) 5 ºC, (b) 20 ºC, (c) 40 ºC, (d) 60 ºC. Dealloyed with 1 M NaOH for 3000 s

In order to isolate the exact position of the scattering knees, the raw scattering data were normalized by dividing each curve by the first acquired data, called $I_0(q)$ (Figure S8). This $I_0(q)$ was obtained for a sample at the very moment just upon contacting with the dealloying solution. The normalized relative intensity I'(q) was again plotted against the logarithmic scattering vector q in Figure S8. This divisional normalization was selected rather a direct background subtraction since the intensity of the visible broad knees was too weak to properly identify the mean distributions. Trials of subtractions were performed, but the signal to noise ratio was too low to further use the data and obtained qualitative information about the structure and physical distributions. Although normalization by dividing the data increased the knee resolution making them more prominent thus facilitating the position identification, the relative intensity could not obviously be used for fitting and further analysis [5]. This method was therefore solely used for peak location and overall peak width evaluation, which were correlated to the scattering vector to retrofit with the raw data.

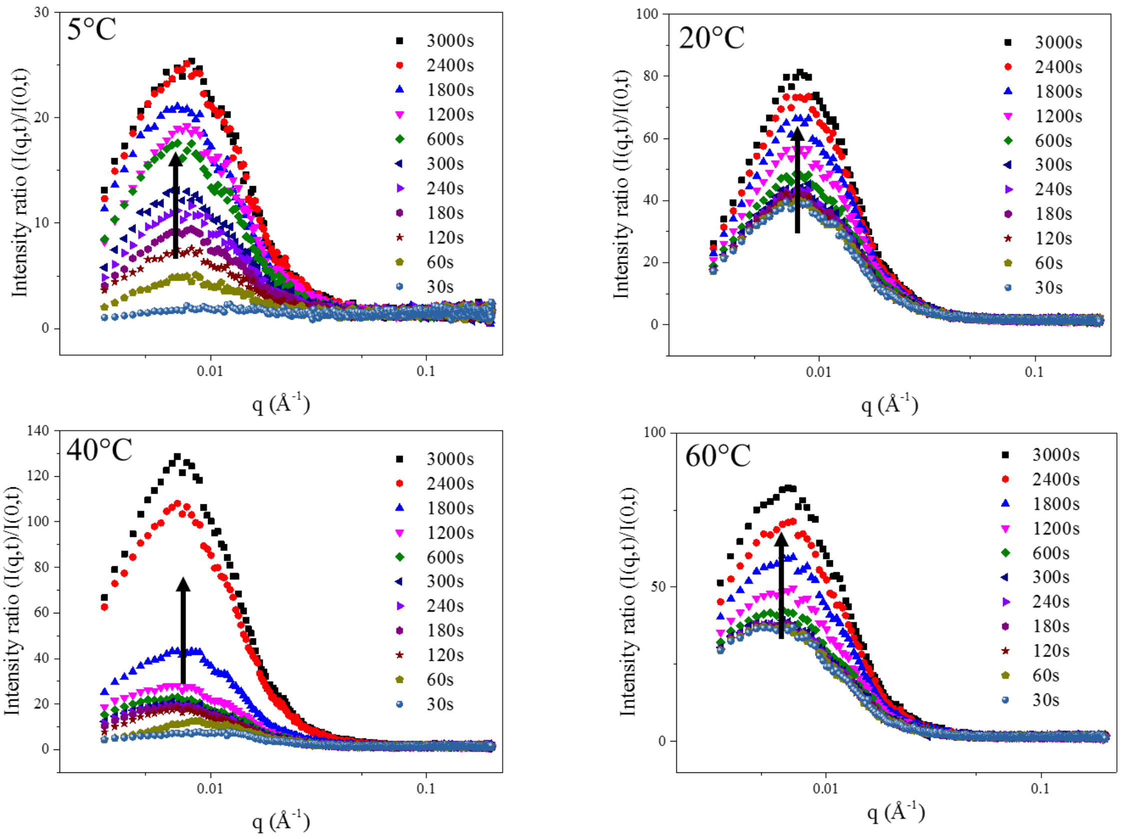

Figure S8 Normalized data curves of in-situ SAXS dealloying test at 5 ºC, 20 ºC, 40 ºC and 60 ºC. The direction of the arrow shows that the intensity of the scattering patterns increased with duration increasing

For the purpose of the SAXS data analysis, the dealloyed metal porous structures were regarded as an amorphous structure [16] since only one broad knee could be identified. Although these knees exhibited a very large range of size distributions, the relatively stable position of the knee centres indicated that these knees represented confidently the dealloyed pores. Thus, the morphology changes of the dealloyed samples over time and for the series of temperatures were investigated through specific peak analysis within the following section.

The X-ray beam was scattered at the interface between the different phases, including the ligaments and the solution, but also the CuO precipitates and the brass alloy at scattering vectors $q < 1\ nm^{-1}$. The scattering contrast difference ($\Delta\rho^2$) between Cu (ρ_{Cu}=6.47e^{-5}) and Zn (ρ_{Zn}=5.27e^{-5}) within the alloy is so small that it is hardly distinguishable with SAXS at that high beam energy, far from the fluorescent edge of the materials. Likewise, the contrast difference between the main oxide layer interface CuO (ρ_{CuO}=4.73e^{-5}) and the brass (CuZn30) material (ρ_{brass}=6.15e^{-5}) was low, making any differentiations unwise. Meanwhile, the contrast between the etching solution ($\rho_{1M\ NaOH}$=8.9185e^{-6}) and the average ligament (CuO, Cu or brass) was obviously much larger. Since the density difference between the solid components (Cu, Zn, brass and CuO) is not significant enough, all the scattering beam energy in this experiment was therefore considered as a direct contribution from the interface between the etching solution and the ligaments generated during the dealloying process. Therefore, the knee visible across the reduced data was only correlated in the following section to such interface and features irrespective of the exact material composition. The intensity of the knees and their change as a function of the treatments therefore reveals the amount or density of the scatterers while the width of the knee could be correlated to the distribution of the scattering features. This interpretation was also possible since each sample tested was its own reference. Indeed, the data could be compared directly to the same sample before starting dealloying process, which was taken as the first data curve just upon liquid contacting the surface.

In this section, the normalized curves were used instead of raw curves. As shown in Figure S9, most of the knees were not fully displayed across the q range window. Indeed, it is likely that the distribution of the knees went beyond the 400-nm limit, corresponding to the maximum feature size visible from the q range at 7 m camera length. Therefore, each curve was fitted with a Gaussian fitting function prior to peak analysis to evaluate more precisely the entire size distribution, as shown in Figure S9. The scattering size distribution of dealloyed sample (q range) corresponds to the projected distance distribution of neighbouring interfaces of

dealloyed sample (physical size). These kind of distribution was thought as a random variable continuous distribution, which can theoretically be fitted with Gaussian function [17].

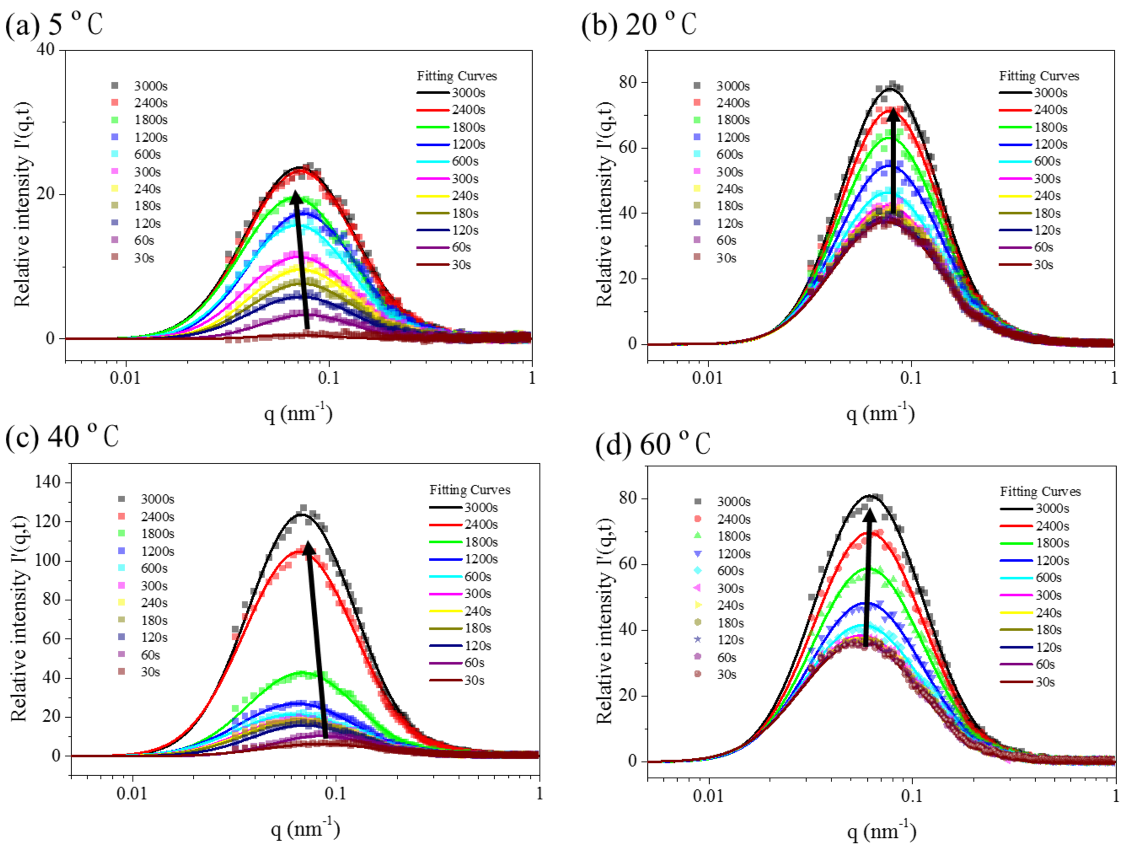

Figure S9 Extended normalized data curves of in-situ SAXS dealloying test at 5 ºC, 20 ºC, 40 ºC and 60 ºC. The direction of the arrow shows that the intensity of scattering patterns increased with duration increasing

S6. The thickness of oxide layer

The impact of the solution temperature on the dealloying kinetics was assessed in this section. The temperature is used as a dynamic parameter which tends to affect the reaction rate rather than the equilibrium of the reaction.

The comparison of a dealloyed sample fabricated at different temperature is shown in Figure S10. An increase of the solution temperature led to a larger pore penetration depth with an initial 4-fold increase between 5 °C and 20 °C, which are respectively 0.23±0.05 μm and 0.87±0.05 μm respectively, prior to a progressive plateauing above 60 °C up to 80 °C, which are respectively 1.17±0.05 μm and 1.23±0.05 μm. The equivalent average pore size increased by 50 folds between 5 °C and 80 °C, which are respectively 0.04±0.01 μm and 1.15±0.33. The 5 °C, 20 °C and 60 °C dealloyed sample morphologies (dealloyed for 1 h) are shown in Figure 1 (a~c).

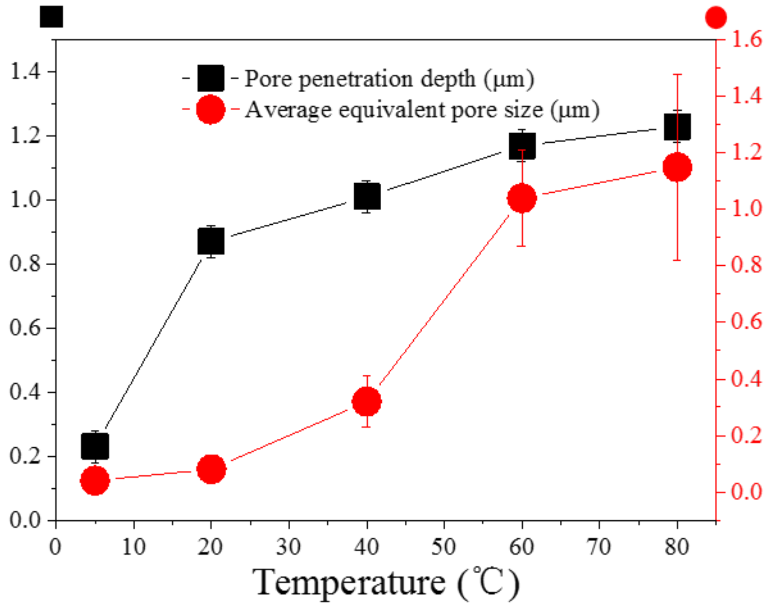

Figure S10 The pore penetration depth and average equivalent pore size of time-based series samples. Dealloyed with 1 M NaOH for 1 h. From SEM analysis. Data from SEM analysis

Long term dealloying experiments of up to 96 h were performed to evaluate the morphologies of dealloyed samples fabricated at different temperature (Figure 1 (d) ~(f)). Compared to room temperature (20 °C), dealloying at low temperature (5 °C) lead to a dual level nano-porous structure. This structure is very similar to that obtained after 51 h dealloyed

morphology at room temperature. The top layer is made of separated fragments while the lower layers compose a continuous nano-porous framework. In addition, the cross-section view of 5 °C dealloyed samples revealed that over 10 consecutive porous layers could be obtained, about 5 times more than that found for the 20 °C dealloyed sample, where only 2 layers were found after 96 h of treatment.

On the other hand, the surface of the 60 °C dealloyed samples is composed of micron-sized precipitates. Cross-section views however revealed that that these precipitates are interconnected (Figure 1 (f)) and that their penetration depth (the darker area of sample) is around 2 μm. This penetration depth is smaller than that obtained for the 20 °C dealloyed sample, which is approximate 5 μm at 96 h.

The results indicate that increasing solution temperature led to higher reaction rates. Although the 5 °C dealloyed sample offered smaller penetration depth and the highest ratio of residual Zn, this temperature led to the smallest average pore size (20 nm) and narrowest size distributions (± 0.01 μm). In addition, there were no precipitated particle across the 5 °C dealloyed samples, while the precipitated particles appeared at a higher temperature (40°C, 60 °C and 80 °C). The size of these precipitates was also found to increase with solution temperature increasing. The smaller penetration depth for the 60 °C dealloyed sample after 96 h of treatment may be caused by premature CuO precipitation, which was dissolved faster at that temperature compared to that at room temperature. The less exposed surface of the pristine matrix may have therefore led to slower reaction rate. However, although the dealloying process progressed slowly at high dealloying temperature, the dramatic change of morphology with dealloying process progressing revealed that the activity of dealloyed surface at higher dealloying temperature remained significant.

An increase of the solution temperature did not only accelerate the process but also changed the morphology of the products and passivated the surface of the samples. It implied that the precipitation process is an independent sub-process of the dealloying process. Thus, the dealloying process is combined by the competitive etching process and the precipitating process. At the former process, both Cu and Zn will be etched by etching solution but Zn prior to be etched, while dissolved Cu will be precipitated from solution and deposit onto the surface of the samples during the latter process. The difference in sensitivity of temperature between

these two processes let to the different morphology of products. The precipitation process and routes to remediate it will be discussed in following section.

Examples of RG fits at the different temperatures are provided in Figure S11.

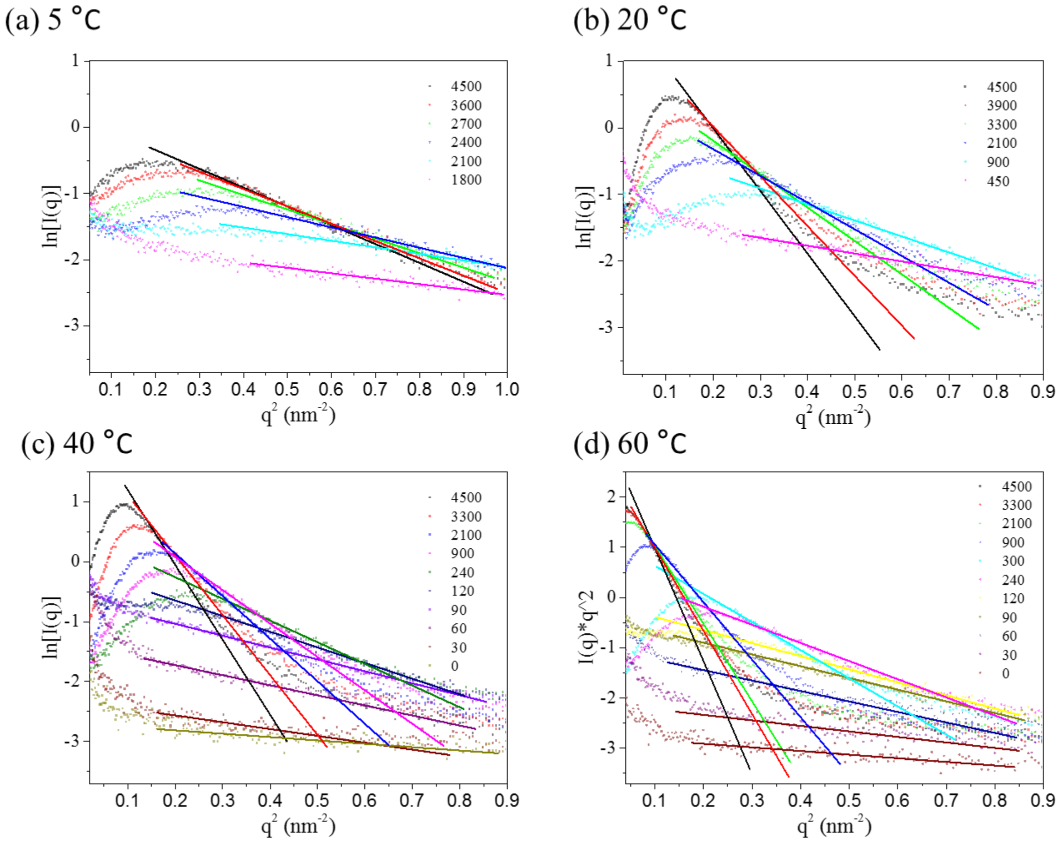

Figure S11 Illustration of R_g fitting of Au-Ag SAXS patterns at different temperatures

S7. Additional schematics and figures

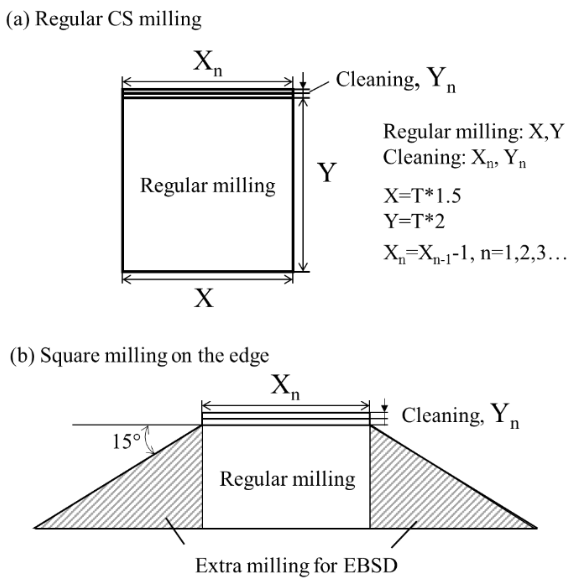

(c) Ion beam current and Y dimension for each milling section

		Current	$Y_{(n)}$
Regular milling		7 nA	2*T
Cleaning	1	1 nA	2
	2	0.5 nA	0.5
	3*	50 pA	0.4

* Cleaning 3 was applied for cleaning CS of de-alloyed sample.

Figure S12 Schematic of CS milling with FIB. (a) Pattern size of regular CS milling. (b) Pattern size of CS/EDS/EBSD milling on the edge. (c) Ion beam current and Y dimension for each milling section

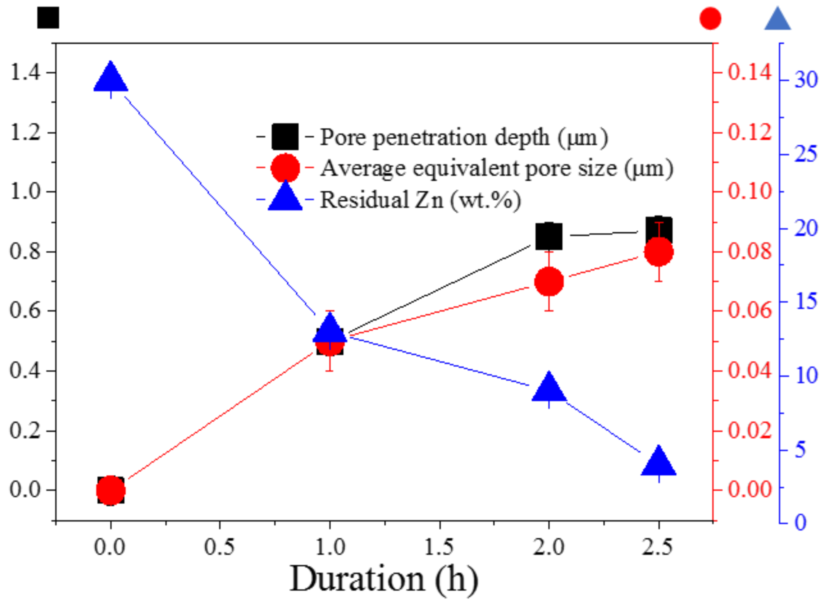

Figure S13 The pore penetration depth, average equivalent pore size and residual Zn of time-based series samples. De-alloying with 1 M NaOH at room temperature. Data extracted from SEM analysis and published at [18]

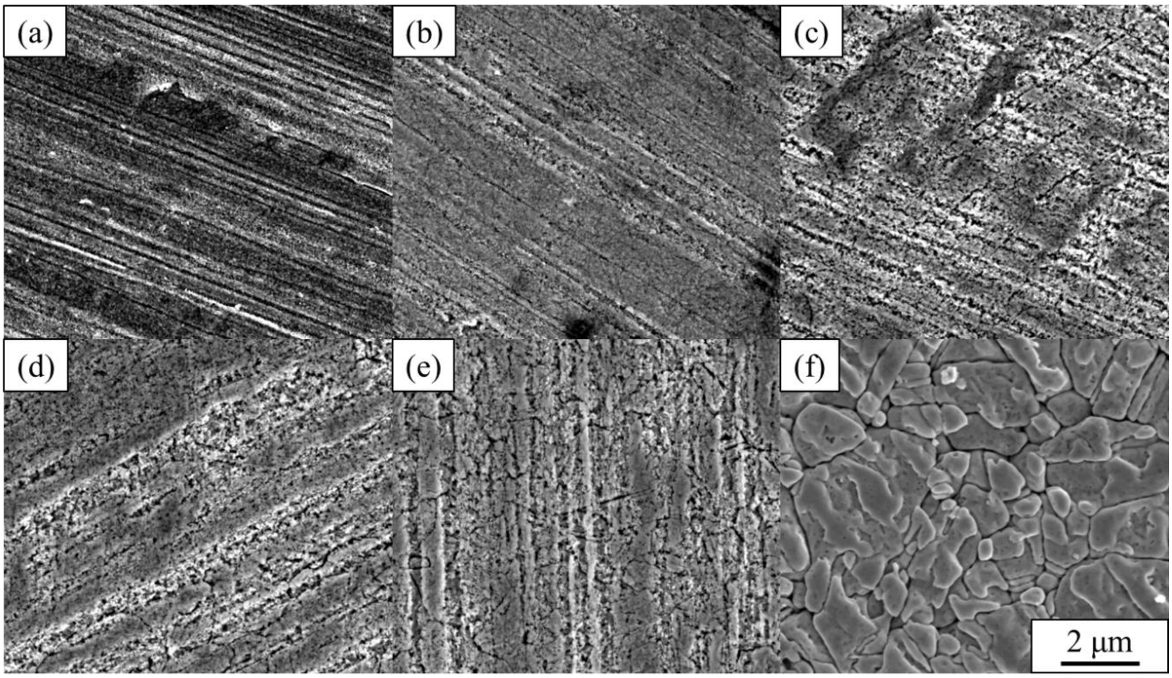

Figure S14 The SE images of samples dealloyed with 1M NaOH at room temperature. (a) Dealloying for 1 h. (b) dealloying for 2 h. (c) dealloying for 2.5 h. (d) dealloying for 3 h. (e) dealloying for 5 h. (f) dealloying for 51 h

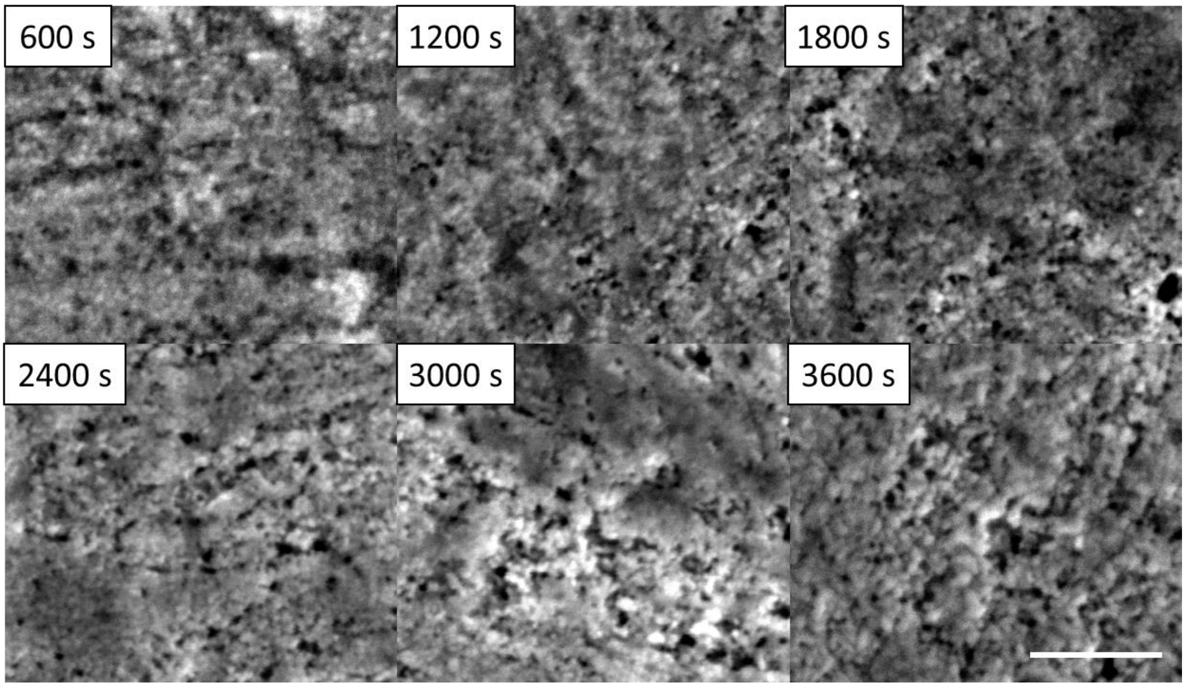

Figure S15 The SE images of dealloyed Cu-Zn sample. Dealloyed with 1 M NaOH at 5 °C. Scale bar: 500 nm

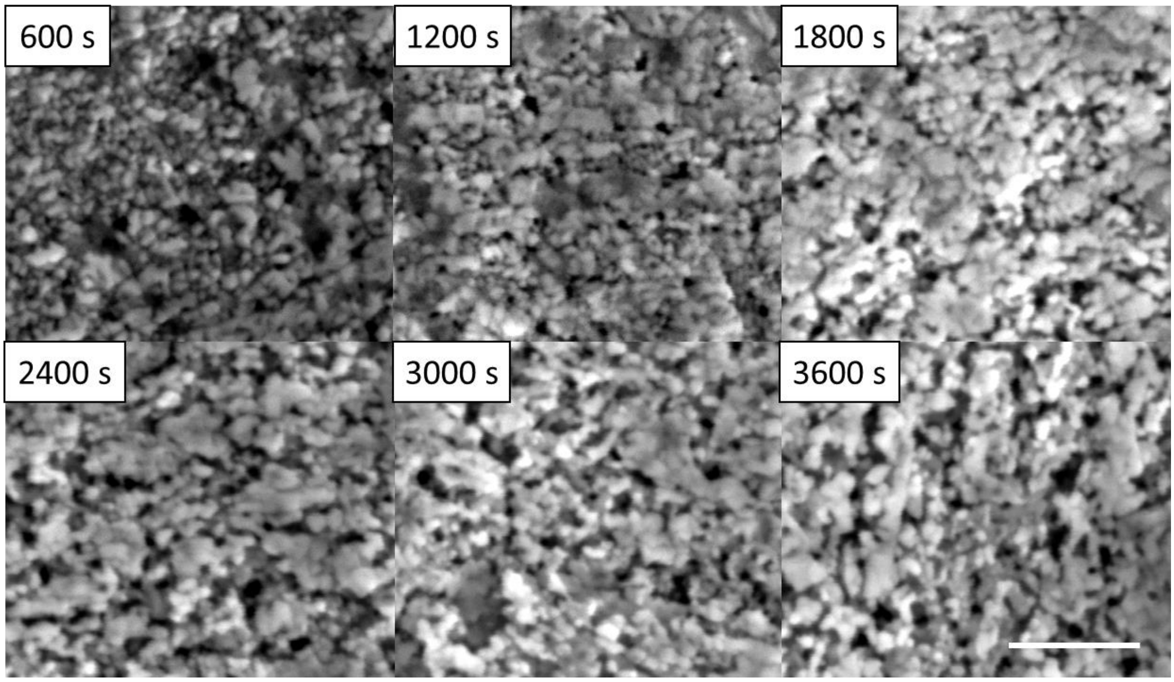

Figure S16 The SE images of dealloyed Cu-Zn sample. Dealloyed with 1 M NaOH at 20 °C. Scale bar: 500 nm

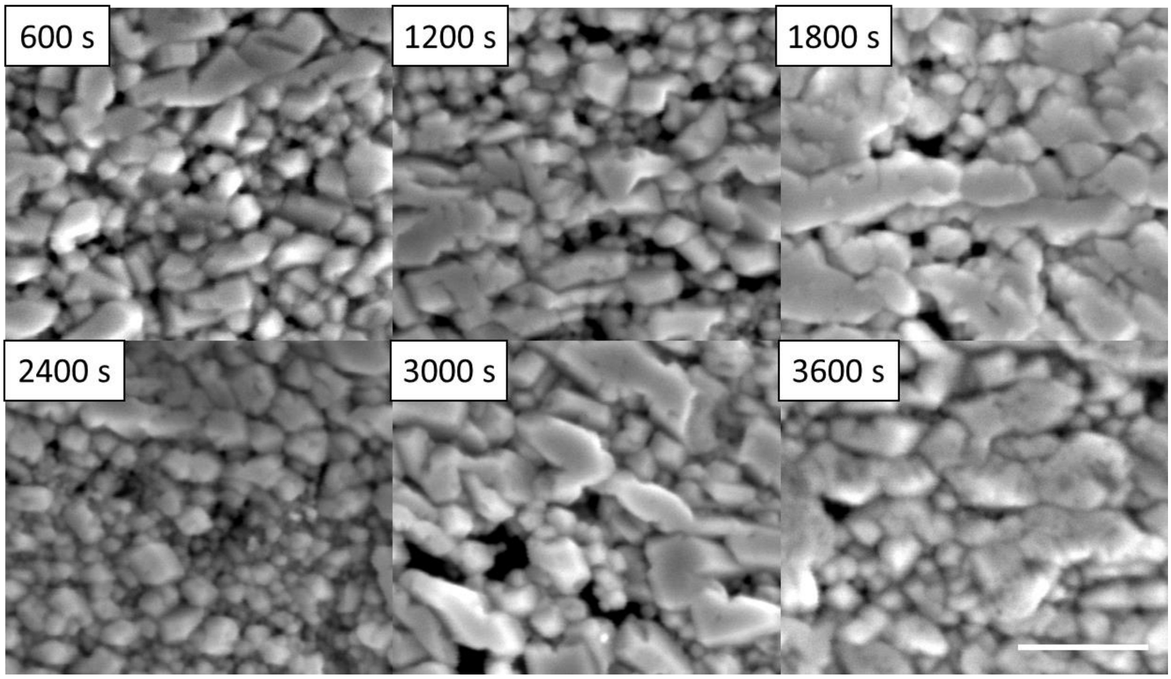

Figure S17 The SE images of dealloyed Cu-Zn sample. Dealloyed with 1 M NaOH at 40 °C. Scale bar: 500 nm

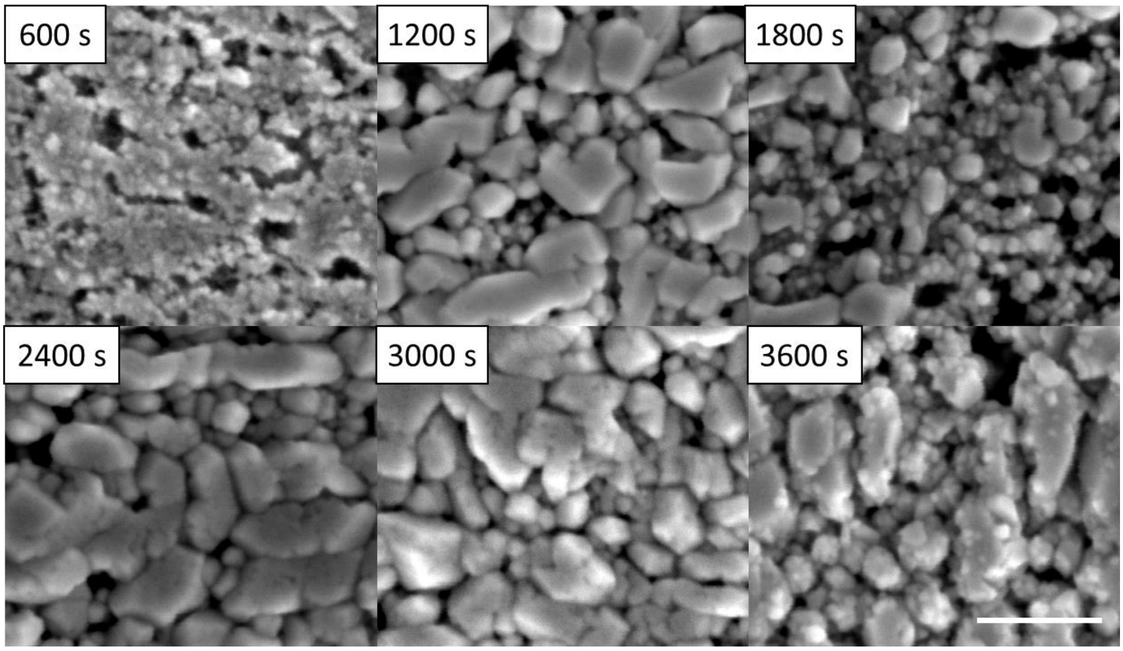

Figure S18 The SE images of dealloyed Cu-Zn sample. Dealloyed with 1 M NaOH at 60 °C. Scale bar: 500 nm

References

1 Gertsman, V. Y. & Bruemmer, S. M. Study of grain boundary character along intergranular stress corrosion crack paths in austenitic alloys. *Acta Materialia* **49**, 1589-1598, doi:http://dx.doi.org/10.1016/S1359-6454(01)00064-7 (2001).

2 Kitagawa, K. On the development of the (001) texture of gold leaf fabricated by hammering. *J Mater Sci* **23**, 2810-2814 (1988).

3 Parida, S. *et al.* Volume Change during the Formation of Nanoporous Gold by Dealloying. *Physical Review Letters* **97**, 035504 (2006).

4 Gallas, J. M., Littrell, K. C., Seifert, S., Zajac, G. W. & Thiyagarajan, P. Solution Structure of Copper Ion-Induced Molecular Aggregates of Tyrosine Melanin. *Biophysical Journal* **77**, 1135-1142, doi:http://dx.doi.org/10.1016/S0006-3495(99)76964-X (1999).

5 Fink, H. P. Structure analysis by small-angle X-ray and neutron scattering. Von LA FEIGIN und DI SVERGUN. ISBN 0-306-42629-3. New York/London: Plenum Press 1987. XIII, 335 S., geb. US $95.40. *Acta Polymerica* **40**, 224-224 (1989).

6 Glatter, O. & Kratky, O. *Small angle X-ray scattering*. (Academic press, 1982).

7 Hu, N., Borkar, N., Kohls, D. & Schaefer, D. W. Characterization of porous materials using combined small-angle X-ray and neutron scattering techniques. *Journal of Membrane Science* **379**, 138-145 (2011).

8 Putnam, C. D., Hammel, M., Hura, G. L. & Tainer, J. A. X-ray solution scattering (SAXS) combined with crystallography and computation: defining accurate macromolecular structures, conformations and assemblies in solution. *Quarterly reviews of biophysics* **40**, 191-285 (2007).

9 Salinas-Nolasco, M. F. & Méndez-Vivar, J. Correlation between Fractal Dimension and Surface Characterization by Small Angle X-ray Scattering in Marble. *Langmuir* **26**, 3889-3893, doi:10.1021/la903835m (2010).

10 Sinha, S., Sirota, E. B., Garoff, S. & Stanley, H. X-ray and neutron scattering from rough surfaces. *Physical Review B* **38**, 2297 (1988).

11 Gad-Allah, A., Abou-Romia, M., Badawy, M. & Rehan, H. Passivity of α-brass (Cu:Zn/67:33) and its breakdown in neutral and alkaline solutions containing halide ions. *Journal of applied electrochemistry* **21**, 829-836 (1991).

12 Dinnappa, R. & Mayanna, S. The dezincification of brass and its inhibition in acidic chloride and sulphate solutions. *Corrosion science* **27**, 349-361 (1987).

13 Wang, J., Jiang, X. & Li, S. Synergistic mechanism of boron and arsenic in preventing dezincification of brass. *Chinese Science Bulletin* **42**, 1135-1139 (1997).

14 Yun, Q. *et al.* Chemical Dealloying Derived 3D Porous Current Collector for Li Metal Anodes. *Advanced Materials* (2016).

15 Cahn, R. W. Materials science: Percolation frustrated. *Nature* **389**, 121-122 (1997).

16 Dotzler, C. J. *et al.* In Situ Observation of Strain Development and Porosity Evolution in Nanoporous Gold Foils. *Advanced Functional Materials* **21**, 3938-3946 (2011).

17 Casella, G. & Berger, R. L. *Statistical inference*. Vol. 2 (Duxbury Pacific Grove, CA, 2002).

18 Lin, B., Kong, L., Hodgson, P. D. & Dumée, L. F. Impact of the De-Alloying Kinetics and Alloy Microstructure on the Final Morphology of De-Alloyed Meso-Porous Metal Films. *Nanomaterials* **4**, 856-878 (2014).
